# Partial defoliation of *Brachypodium distachyon* plants grown in petri dishes under low light increases P and other nutrient levels concomitantly with transcriptional changes in the roots

**DOI:** 10.7717/peerj.7102

**Published:** 2019-06-13

**Authors:** Wei Wang, Sunil Kumar Singh, Xiwen Li, Hui Sun, Yu Yang, Min Jiang, Hailing Zi, Renyi Liu, Huiming Zhang, Zhaoqing Chu

**Affiliations:** 1 Shanghai Key Laboratory of Plant Functional Genomics and Resources, Shanghai Chenshan Botanical Garden, Shanghai, China; 2 Shanghai Chenshan Plant Science Research Center, Chinese Academy of Sciences, Shanghai, China; 3 Shanghai Center for Plant Stress Biology and Center for Excellence in Molecular Plant Sciences, Chinese Academy of Sciences, Shanghai, China; 4 College of Life Sciences, Shanghai Normal University, Shanghai, China; 5 Center for Agroforestry Mega Data Science and FAFU-UCR Joint Center for Horticultural Biology and Metabolomics, Haixia Institute of Science and Technology, Fujian Agriculture and Forestry University, Fuzhou, Fujian, China

**Keywords:** *Brachypodium distachyon*, Transcriptome, Defoliation, Grass, Nutrition

## Abstract

**Background:**

There have been few studies on the partial defoliation response of grass. It has been unclear how partial defoliation may affect roots at the levels of nutrient accumulation and transcriptional regulation. Hereby we report a comprehensive investigation on molecular impacts of partial defoliation by using a model grass species, *Brachypodium distachyon*.

**Results:**

Our Inductively Coupled Plasma Mass Spectrometry analyses of *B. distachyon* revealed shoot- and root-specific accumulation patterns of a group of macronutrients including potassium (K), Phosphorus (P), Calcium (Ca), Magnesium (Mg), and micronutrients including Sodium (Na), iron (Fe), and Manganese (Mn). Meanwhile, our genome-wide profiling of gene expression patterns depicts transcriptional impacts on *B. distachyon* roots by cutting the aerial portion. The RNAseq analyses identified a total of 1,268 differentially expressed genes in *B. distachyon* with partial defoliation treatment. Our comprehensive analyses by means of multiple approaches, including Gene Ontology, InterPro and Pfam protein classification, KEGG pathways, and Plant TFDB, jointly highlight the involvement of hormone-mediated wounding response, primary and secondary metabolites, and ion homeostasis, in *B. distachyon* after the partial defoliation treatment. In addition, evidence is provided that roots respond to partial defoliation by modifying nutrient uptake and rhizosphere acidification rate, indicating that an alteration of the root/soil interaction occurs in response to this practice.

**Conclusions:**

This study reveals how partial defoliation alters ion accumulation levels in shoots and roots, as well as partial defoliation-induced transcriptional reprogramming on a whole-genome scale, thereby providing insight into the molecular mechanisms underlying the recovery process of grass after partial defoliation.

## Introduction

Defoliation by means of cutting causes mechanical damage (MD) to plants, which need to respond quickly and to adapt to such a challenge. Defoliation by insect herbivory (HD) or MD led to a rapid and transient reduction of non-structural carbohydrates in all tissues examined (Castrillon-Arbelaez et al., 2012). The physiological and biochemical responses to increasing levels of mechanical leaf removal up to total defoliation were quantified. Tolerance appeared to be dependent on various factors: (i) amount of lost tissue; (ii) mechanics of leaf tissue removal; (iii) environment, and (iv) species tested (Vargas-Ortiz et al., 2013). The biochemical processes underlying partial defoliation are often accompanied by source-sink transitions affecting carbon (C) metabolism, but could not compensate when defoliation occurred during flowering (Vargas-Ortiz, Délano-Frier & Tiessen, 2015). In plants, the defense network in response to wounding largely overlaps with the responses activated by pathogen attack and herbivore injury (Reymond et al., 2000; Arimura, Kost & Boland, 2005; Rehrig et al., 2014). Plant responses to stress conditions may be classified into two stages. The first stage is known as the sensory/activation stage, followed by the second stage, that is, physiological stage, during which plants respond to the perceived stress (Priest et al., 2014). Perception of stress cues can be followed by actions of secondary messengers such as calcium and inositol phosphates, as well as by production of reactive oxygen species (ROS) (Zhu, 2001; Priest et al., 2014) and other signaling molecules such as ethylene (ET) and jasmonic acids (JA) (Turner, Ellis & Devoto, 2002; Koo & Howe, 2009; Jacobo-Velázquez, González-Agüero & Cisneros-Zevallos, 2015). The Ca^2+^ ions further initiate various phosphorylation cascades mediated by Ca^2+^-binding proteins which subsequently affect different transcription factors (TF), resulting in the production of stress-responsive proteins and secondary metabolites to combat the wounding stress. For example, carrots produce some phenolic compounds such as caffeoylquinic acids at site of wounding in response to the stress (Jacobo-Velázquez et al., 2011). Likewise, synthesis of nicotine is stimulated by mechanical wounding in tobacco (Xi, Li & Zhang, 2008). As a common practice for grass or forage crops, defoliation causes severe MD to plants, yet how defoliation impacts roots at the molecular level on a whole-genome scale remains to be elucidated.

For grass or forage crops, defoliation by mowing or by animal grazing is a common practice, which removes a significant portion of the above-ground parts of plants for managing grassland or for feeding ruminants. Defoliation drastically impacts plant growth because it removes leaves that are the photosynthesis active organ (Ludlow & Charles-Edwards, 1980; Parsons et al., 1983). After defoliation, the demand of energy for subsequent growth of new leaves and for respiration is far from being fulfilled by the remaining photosynthesis activity; instead, the energy demand is met by utilization of starch and mobilization of water-soluble carbohydrates such as glucose, fructose, sucrose, and fructans, from the reserves in the remaining stubbles (Donaghy & Fulkerson, 1997; Morvan-Bertrand et al., 2001). It has been demonstrated that the nutrients mobilized are mainly utilized by growing leaves (Prud’homme et al., 1992; De Visser, Vianden & Schnyder, 1997). The effect of defoliation is also evident by cessation or reduced root growth (Davidson & Milthorpe, 1966; Ryle & Powell, 1975; Jarvis & Macduff, 1989). Several studies have been conducted to investigate the effects of defoliation on plant nitrate uptake (Millard, Thomas & Buckland, 1990; Thornton & Millard, 1996, 1997). In contrast, it has been unclear how defoliation may influence homeostasis of metal ions, such as potassium (K^+^) and calcium (Ca^2+^) that are essential nutrients, in plants.

Grasses are important forage and horticulture plants. The introduction of *Brachypodium distachyon* as a model species for grass family by Draper et al. (2001) has greatly facilitated unraveling the unique nature of grass species in response to different environmental cues. In 2010, the genome of *B. distachyon* inbred line 21 (Bd21) was reported as the first mapped genome of the Pooideae family (International Brachypodium Initiative, 2010). Since then, lots of studies have been carried out with grass species to investigate various questions, such as the effects of partial defoliation on soil surface temperature and moisture (Wan, Luo & Wallace, 2002), availability of light to plants (Parr & Way, 1988), CH_4_ uptake in relation to soil microbial growth (Zhou et al., 2008; Gavrichkova et al., 2010) and CH_4_ uptake fluxes (Zhang et al., 2012). Cruz (1997) reported that effect of shade on the growth and mineral nutrition of a C4 perennial grass under field conditions, Kitchen, Blair & Callaham (2009) reported that annual fire and mowing altered biomass, depth distribution, and C and N content of roots and soil in tallgrass prairie and Eom et al. (1999) reported the effect of fire, mowing and fertilizer amendment on arbuscular mycorrhizas in tallgrass prairie. In contrast, genome-wide transcriptional impact of partial defoliation on grass roots has been unclear. It has also been unclear how partial defoliation may alter root ion uptake of soil nutrients and their whole-plant accumulation patterns. Nutrient uptake by roots can be affected by rhizosphere acidity, because of the electron charges across the root plasma membrane need to be balanced, and because rhizosphere acidification by root proton exudation can increase solubility and therefore availability of some nutrients such as phosphorus in calcareous soil (Rao et al., 2002). It is also unknown whether partial defoliation can affect other root characteristics such as rhizosphere acidification in grass plants. In this study, we investigated the impacts of partial defoliation on *B. distachyon*. Our inductively coupled plasma mass spectrometry (ICP-MS) analyses revealed shoot- and root-specific accumulation patterns of a group of macronutrients including potassium (K), Phosphorus (P), Calcium (Ca), Magnesium (Mg), and micronutrients including Sodium (Na), iron (Fe), and Manganese (Mn) accompanied by reduced rhizosphere acidification by *B. distachyon* roots. Meanwhile, our genome-wide profiling of gene expression patterns depicts transcriptional impacts on *B. distachyon* roots by cutting the aerial portion.

## Materials and Methods

### Plant growth conditions

The grass species *B. distachyon* was used in this study. Seeds were surface sterilized by 75% ethanol for 5 min, then by 10% NaClO for 30 min, and were washed three times by sterilized distilled water. The seeds were then subjected to imbibition for another 12 h before sown in sterilized transparent plastic boxes (5 × 10 × 8 cm), which contain 200 mL 1/2-strength Murashige and Skoog *medium* (MS medium) with 1% sucrose and 0.65% agar, pH adjusted to 5.7. A total of 20 seeds were sown in each box. Seedlings in the boxes were grown in a plant growth chamber at 23 °C, 55% humidity, and 75 μmol·m^2^·s^−1^ light intensity under a 16-h light/8-h dark photoperiod.

### Leaf-cutting treatment and sample harvesting

For leaf-cutting assay, 2-week-old seedlings grown in each box were divided into two halves, one for treated and the other for control. Pruned seedlings were cut by scissors and 1/3 stub was left for each plant (Fig. S12). The remaining half in the same box was used as control plants. After treatment, plants were allowed to continue growth as indicated in the Figures and Tables before being harvested for further analyses.

### ICP-MS analysis of ion contents

A total of 2-week-old *B. distachyon* plants were pruned as described above. Plant shoots and roots were separately collected, and then washed with deionized double-distilled water (DD H_2_O) for four times and dried at 70 °C in an oven for 3 days. After cooling, 5–10 mg dried plant samples were digested with five mL nitric acids at 140 °C in a digestion block for 1.5 h. The digests were diluted 10-folds with sterile DD H_2_O and then subjected to element measurements including Na, Mg, P, K, Ca, Mn, and Fe by using ICP-MS (NexION 300D; PerkinElmer, Waltham, MA, USA). Ion concentrations were calculated based on sample dry weights. Blank samples were run before and after the plant tissue samples to ensure the cleanness of the detection system. Indium as an standard reference materials was used and the recovery rate was between 99% and 105%. Values are mean ± SD, *n* ≥ 3 biological replicates. Asterisks indicate statistical significance, student *t*-test, *p* < 0.05.

### Gene expression profiling by RNAseq

A total of 2 days after treatment (DAT), Trizol reagent (15596; Ambion, Suwanee, GA, USA) was used to isolate total RNA from roots of intact and pruned plants. RNA was treated with DNase (Turbo) to remove any genomic DNA contamination. Each sample has three biological replicates. Library construction and deep sequencing were performed by the Core Facility of Genomics in Shanghai Center for Plant Stress Biology, China. The sequence data have been deposited in NCBI’s Gene Expression Omnibus (GEO) and are accessible through GEO Series accession number GSE101500.

The libraries were sequenced on Illumina Genome Analyzer II and 150 bp paired-end reads (R1 and R2) were produced. We used FastQC (Andrews, 2010) to perform quality control checks and found the R2 reads in two treatment samples (T-2 and T-3) were of low quality (Fig. S4) while both R1 and R2 reads from other samples were of excellent quality, therefore we only used R1 reads for T-2 and T-3 samples and used paired-end reads for other samples to do subsequent analysis. We used SolexaQA and cutadapt to remove low quality regions and adapter sequences from the raw reads and the resulted clean reads with length larger than 25 nt and a Phred quality score larger than 17 were aligned to the genome of *B. distachyon* from phytozome v11 (International Brachypodium Initiative) using Tophat2.01 with default parameters (Chen, Lun & Smyth, 2014). Two mismatches were allowed for each read and both ends with reasonable distance were considered a good mapping result when using paired-end reads as input. The rate of unique mapping all reached to 80% in all samples (Fig. S10). The uniquely mapped reads were selected for gene counting. The correlation coefficients between replicates of each sample are more than 99% (Fig. S11) and the replicates are clustered in the PCA graph (Fig. S4). Differential expression analysis was performed using edgeR 3.8.6 (Kim et al., 2013). Differentially Expressed genes (DEGs) are defined as fold change ≥ 1.5 and FDR ≤ 0.05. DEGs were conducted with gene ontology (GO) enrichment analysis (Alexa, Rahnenfuhrer & Lengauer, 2006). GO terms with *p*-value ≤ 0.001 were selected as significant enriched. Orthologs identification was performed by using the web server viz. g:profiler (Reimand et al., 2016). TF target gene identification was performed by using the TF enrichment function of the web server-Plant Transcriptional Regulatory Map (Jin et al., 2017).

### Rhizosphere acidification detection

Rhizosphere pH change in the medium was determined by a pH indicator visualization assay (Yi & Guerinot, 1996). The medium contains 1/2-strength MS medium with 1% sucrose and 0.7% agar, 0.2 mM CaSO_4_ and the pH indicator bromocresol purple (0.006%). The pH of the medium was adjusted to 6.5 with NaOH. *B. distachyon* plants were initially grown in medium without pH indicator and were treated as described above. At 1, 2, and 3 days after the cutting treatments, plants were transferred to the medium with pH indicator for 24 h before taking pictures. Quantification of rhizosphere acidification was performed according to Yao & Byrne (2001).

### Quantitative real time PCR analysis

Total RNA (50–100 mg) was isolated from root and shoot of *B. distachyon* using RNAzol^®^RT (MRC, Cincinnati, OH, USA) following manufacturer’s instruction. RNA sample were quantified using NanoDrop 2000 (Thermo Scientific Inc., Waltham, MA, USA) and stored at −80 °C till further use. Total RNA (1.0 μg) was converted to ss-cDNA using Transcript one-step gDNA removal and cDNA synthesis Super mix (Transgen, Beijing, China) as per manufacturer’s protocol. qRT-PCR reactions were performed in BIO-RAD C1000 Touch™ Thermal cycler system using iTaq™ universal SYBR^®^ green Supermix qPCR Kit (BIO-RAD, Hercules, CA, USA). Total cDNA was diluted to ∼25 ng/μL and a total 100 ng was used in a 20 μL reaction mixture. For each reaction, three technical replicates were used along with no template control to check for contaminants. The following thermal cycling programme was used for all qRT-PCR reactions: 3 min at 95 °C, 3 s at 95 °C and 45 s at 60 °C for 40 cycles, which includes data acquisition. Finally, a dissociation curve analysis was performed from 65 to 95 °C in increments of 0.5 °C, each lasting for 5 s, to confirm the presence of a specific product. Concentration of ACT2 (At3g18780) was used to normalize the gene expression in different samples. Log fold change in expression values were calculated using the 2^−ΔΔCT^ method (Livak & Schmittgen, 2001).

## Results

### Cutting aerial portions alters nutrient uptake patterns in *B. distachyon*

**Figure 1:**
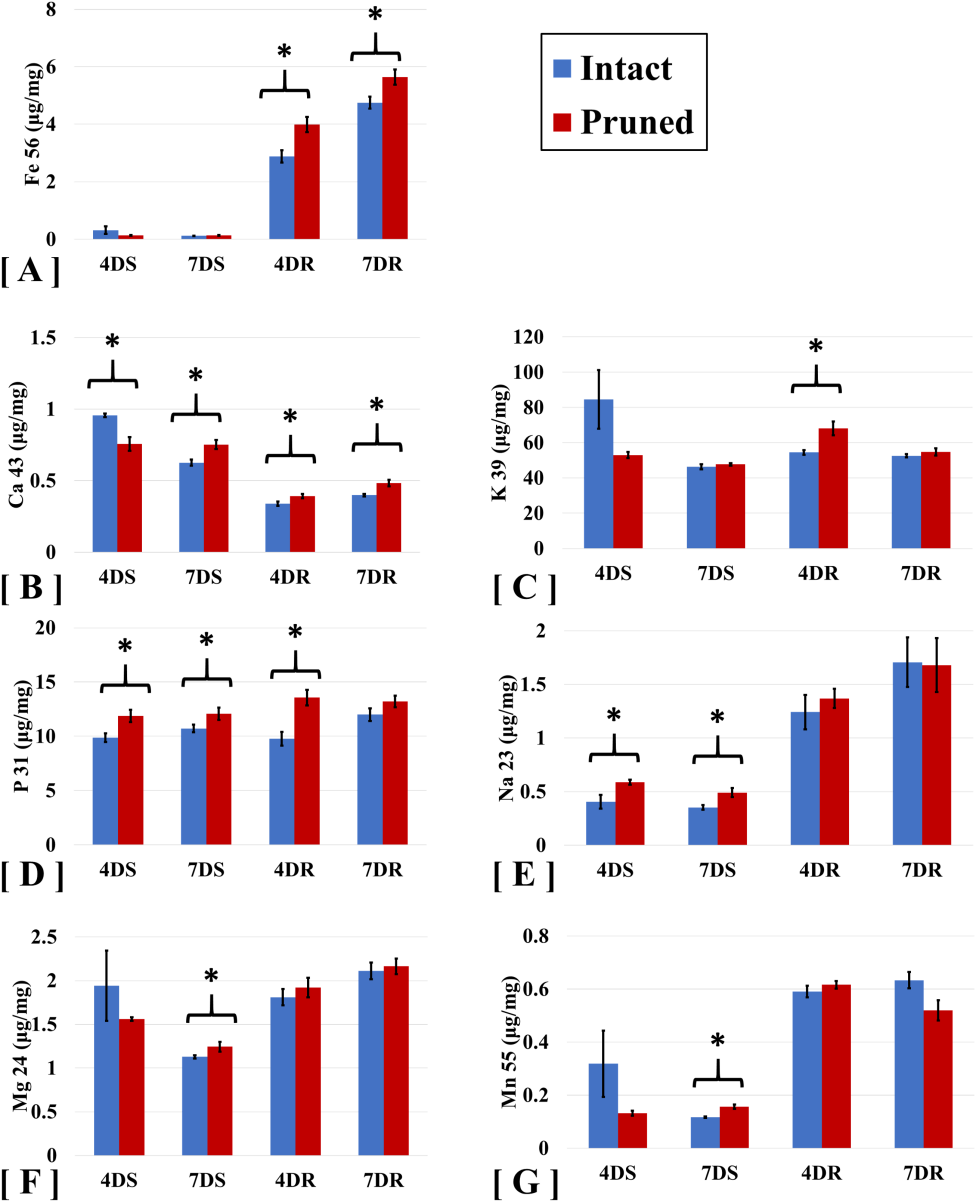
ICP-MS analyses of ion accumulation levels in *Brachypodium distachyon* shoots and roots. Ions analyzed include (A) Iron (Fe), (B) Calcium (Ca), (C) Potassium (K), (D) Phosphorous (P), (E) Sodium (Na), (F) Magnesium (Mg), and (G) Manganese. Samples were collected at 4 and 7 days after treatment (4D and 7D) and separated into shoots (S) and roots (R). Values are mean ± SD, *n* ≥ 3 biological replicates. Asterisks indicate statistical significance, student *t*-test, *p* < 0.05.

To examine the impact of partial defoliation on nutrient uptake in *B. distachyon*, we performed ICP-MS to measure the levels of a group of macronutrients which including K, P, Ca Mg, and micronutrients including Na, Fe, Mn elements in both roots and shoots. Compared to the intact plants, roots of *B. distachyon* with defoliation showed 38% and 19% increases in Fe levels at 4 days and 7 DAT, respectively; whereas Fe levels in shoots were similar between the pruned and the intact plants (Fig. 1A). The elevation in Fe contents in pruned plants thus seems consistent with an increased demand of Fe for restoration of photosynthesis organ after defoliation. Similar to Fe, accumulation of Calcium (Ca) was increased in roots by cutting the aerial portion. At 4 and 7 DAT, the levels of Ca in pruned roots were 13% and 22% greater than those in intact roots (Fig. 1B). Interestingly, defoliation caused a reduction in shoot Ca levels initially at 4 DAT, while at 7 DAT, Ca levels were higher than those in intact plants.

Potassium (K) and phosphorus (P) are two major macronutrients necessary for normal plant growth and development. As expected, these two nutrient elements were detected at levels clearly higher than the other elements examined, including Ca, Fe, Mg, Mn, and Na (Fig. 1). Cutting the aerial portion also elevated P levels by 25% and 43% in the shoots and the roots, respectively, in *B. distachyon* at 4 DAT (Fig. 1D). The pattern of increased P accumulation in pruned plants was maintained at least by the time of 7 DAT, although the difference between the pruned and intact plants became less significant compared to that at 4 DAT.

Our ICP-MS analyses of *B. distachyon* ionome also detected defoliation-induced alteration in the levels of sodium (Na), Magnesium (Mg), and Manganese (Mn) (Table 1). As shown in Fig. 1E, accumulation of Na displayed higher levels in roots compared to those in shoots. Cutting *B. distachyon* leaves did not affect Na accumulation in roots at either 4 or 7 DAT, however, the treatment resulted in higher levels of Na in shoots at both 4 and 7 DAT (Fig. 1E). At 4 days after cutting, shoot Mg and Mn levels seemed to be reduced in the pruned plants, albeit no statistical difference was observed when comparing the pruned with the intact samples; in contrast, the cutting treatment resulted in altered levels of both Mg and Mn with statistical significance at 7 DAT (Figs. 1F and 1G).

**Table 1:** Cutting aerial portions alters nutrient uptake patterns in *B. distachyon*

	Fe 56 (ug /mg)		% change	Ca 43 (ug /mg)		% change	K 39 (ug /mg)		% change	P 31 (ug /mg)		% change	Na 23 (ug /mg)		% change	Mg 24 (ug /mg)		% change	Mn 55 (ug /mg)		% change
	T	C		T	C		T	C		T	C		T	C		T	C		T	C	
4DS	0.14	0.32	−57.15	0.76	0.96	−20.92	51.37	56.39	−8.91	11.89	9.88	20.37	0.59	0.41	44.54	1.56	1.94	−19.66	0.13	0.32	−58.70
7DS	0.14	0.12	12.47	0.75	0.63	20.11	48.30	46.10	4.76	12.07	10.71	12.68	0.49	0.35	39.48	1.24	1.13	10.22	0.16	0.12	34.05
4DR	3.99	2.89	38.26	0.39	0.34	15.86	69.13	53.56	29.06	13.57	9.77	38.83	1.37	1.24	10.28	1.92	1.81	6.17	0.62	0.59	4.37
7DR	5.64	4.75	18.82	0.48	0.40	21.40	55.38	51.69	7.14	13.21	12.00	10.10	1.68	1.71	−1.53	2.16	2.11	2.52	0.52	0.63	−17.87
4 D shoot/root	0.03	0.11	−69.01	1.93	2.83	−31.75	0.74	1.05	−29.42	0.88	1.01	−13.30	0.43	0.33	31.06	0.81	1.07	−24.33	0.21	0.54	−60.43
7 D Shoot/root	0.02	0.03	−5.35	1.55	1.57	−1.06	0.87	0.89	−2.23	0.91	0.89	2.34	0.29	0.21	41.64	0.57	0.53	7.52	0.30	0.18	63.23

**Notes:**

Nutrient element concentrations including Na, Mg, P, K, Ca, Mn, and Fe was measured by ICP-MS analysis. Ion concentrations were calculated based on sample dry weights.

DS, treatment after days of shoots; DR, treatment after days of roots; T, treatments; C, control.

### Partial defoliation strongly impacts *B. distachyon* root transcriptome

To better understand how leaf-cutting affects the below-ground portion of *B. distachyon* at transcriptional level, we profiled root gene expression patterns on a whole-genome scale by performing RNAseq analyses. The transcriptome of intact roots and their pruned counterparts were compared at 2 days after the cutting treatment. A total of 1,268 DEGs were identified with ≥ 2-fold change, demonstrating a strong impact on the roots by cutting the aerial portion. Among all the DEGs, 731 and 537 DEGs are upregulated and downregulated (Fig. S1A). Although the genome of *B. distachyon* has been reported (International Brachypodium Initiative, 2010), detailed information of gene function is still largely limited. In the pruned *B. distachyon*, 55% of the DEGs showed GO annotations (Fig. S1B). Subsequently, all the genes were subjected to GO ontology assignment against the *B. distachyon* genome database (available at GO enrichment database of PlantRegMap), in order to classify them by various biological pathways. Three independent ontological classes were used for classifying the DEGs including biological processes, molecular function, and cellular components (Ashburner et al., 2000). DEGs that had GO annotation can be further classified into 110 significant GO terms (*p* < 0.01) with 72 biological processes, 33 molecular function, and five cellular components (Table S1). The top 20 biological processes GO terms and the top 20 molecular function GO terms are shown in Fig. S1, together with the GO terms of five cellular components.

Consistent with the elevated iron accumulation in pruned *B. distachyon* (Fig. 1A), the GO term “cellular response to iron ion starvation” was identified (Table S1). The enriched GO terms for ion homeostasis and binding also include “potassium ion binding,” “magnesium ion binding,” and “alkali metal ion binding,” which can be correlated with the altered accumulation of these two metal ions and Na^+^ as observed by ICP-MS (Fig. 1).

### Partial defoliation induces transcriptional alterations on plant ion homeostasis, hormones, primary and secondary metabolites, and wound stress

**Figure 2:**
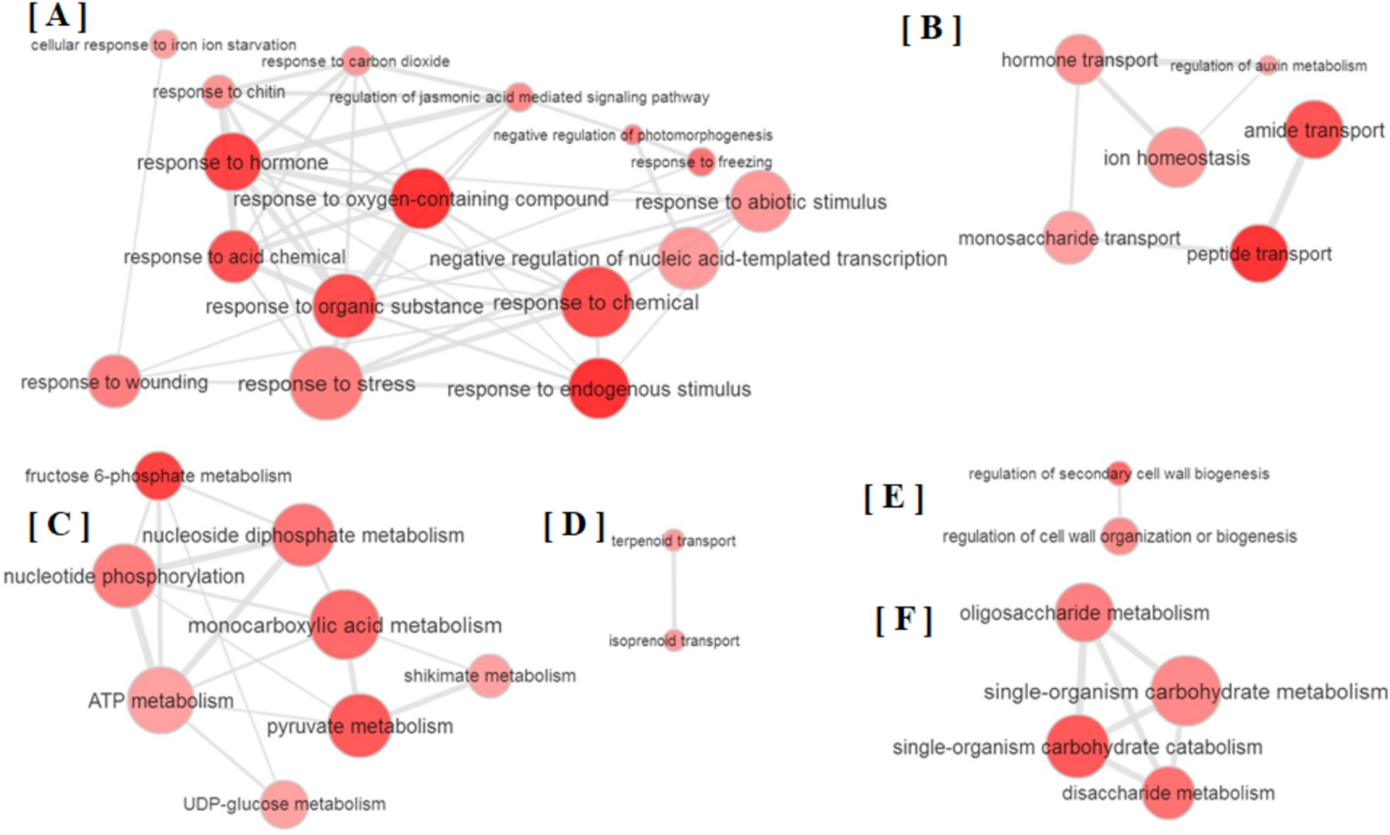
Depiction of major biological processes as revealed by Reduce visualize gene ontology module (REVIGO) for ≥ 2-fold DEGs in *B. distachyon* treated by cutting the aerial portion. Major clustering of GOs pertaining to (A) stress response, (B) ion homeostasis, (C) secondary metabolites, (D) secondary metabolite transportation, (E) regulation of cell biogenesis and (F) primary metabolism.

All the GO terms obtained were further subjected to removal of redundancy by using REVIGO (Supek et al., 2011). The REVIGO analysis for 110 GO terms clearly grouped them into six major clusters (Fig. 2), which reveals that partial defoliation leads to differential expression of genes pertaining to stress and TF (Fig. 2A), hormone mediated ion homeostasis (Fig. 2B), secondary metabolism and transportation of its metabolites (Figs. 2C and 2D), cell organization (Fig. 2E) and primary metabolism (Fig. 2F) in *B. distacyon*. Moreover, these DEGs may further be classified into different gene functional groups based on the shared biological function (Table S2), with the top three most shared function being transcription regulation (60 DEGs), stress response (48 DEGs), and signal transduction (43 DEGs). Together these GO patterns display a complex regulatory network at transcriptional level underlying *B. distachyon* response to the partial defoliation treatment.

Among the DEGs, a group of 48 genes categorized as related to “response to hormone,” in addition to seven DEGs as related to “hormone transport” (Table S1; Fig. S2A). Among these DEGs, most of the gibberellin related DEGs were up-regulated (Table S3). This pattern may suggest that gibberellin-mediated plant growth is a key process during *B. distacyon’s* responses to partial defoliation, since gibberellin is known for its essential role in plant growth such as stem elongation, leaf expansion and trichome development (Davière & Achard, 2013). The involvement of phytohormones in *B. distacyon’s* responses to partial defoliation is also indicated by a subset of DEGs related to phytohormone-mediated wounding responses; these DEGs are associated with auxin (Bradi1g06670, Bradi4g21220), abscisic acid (Bradi4g24120, Bradi5g18830), ET (Table S3), JA (12 DEGs), and JA-mediated signaling (Bradi1g72610, Bradi3g23180, Bradi3g23190, Bradi5g08650).

In addition to showing altered phytohormone gene expression, plants pruned by partial defoliation also displayed transcriptional regulation on DEGs related to homeostasis of secondary metabolites such as terpenoids and isoprenoids (Table S1; Fig. S2B). Plant primary metabolism may also be affected by partial defoliation, because the over-represented DEGs include those related to carbohydrate metabolic process, fructose, sucrose, di and oligo-saccharide metabolism, glycolytic process, pyruvate metabolic process, and glucose import and transport (Table S1; Fig. S2C). Particularly, sucrose synthase activity is the GO term that shows lowest p-value among all GO terms (Table S1). It is also possible that these membrane- or vacuole-related GO terms reflect in planta ion transportation and compartmentalization. Together with the ion accumulation patterns, the overrepresentation patterns of DEGs related to membrane and vacuole, to different transporters, and to ion homeostasis and ion uptake (Table S1; Fig. S2D), collectively suggest that plant responses to partial defoliation include not only elevated uptake of certain nutrients and altered primary and secondary metabolism, but also possibly cellular re-distribution of secondary metabolites and nutrient ions.

According to the InterPro classification, the enriched protein families belong to transporters (IPR020846, IPR013525, and IPR000109) and membrane specific proteins (IPR006016, IPR008004, and IPR005516). These patterns further support the hypothesis of plant transcriptional regulation for elevated uptake of nutrition from the rhizosphere. The protein families belonging to sugar binding (PF14416) and synthesis (IPR000368) reveal changes in energy homeostasis in mowed grasses, while protein families of different TF (like myb, bHLH bBox type, etc), and stress-related proteins (Fig. S3; Table 2) provide further mechanistic clues for plant responses to partial defoliation.

**Table 2:** Enriched InterPro and Pfam protein domains in mowed *B. distachyan* transcriptomes were shown.

#pathway ID	Pathway Description	Observed gene count	Biological significance (Source: InterPro)
IPR025322	Protein of unknown function DUF4228, plant	12	Functionally uncharacterized
IPR006016	UspA	8	A small cytoplasmic protein, enhances the rate of cell survival during stress
IPR011598	Myc-type, basic helix-loop-helix (bHLH) domain	15	Specific DNA-binding proteins act as transcription factor
IPR001005	SANT/Myb domain	19	Nuclear DNA-binding protein
IPR020846	Major facilitator superfamily domain	19	Transporters drive solute accumulation or extrusion by using ATP hydrolysis, photon absorption, electron flow, substrate decarboxylation or methyl transfer, generates electron chemical potential in case of consumption of primary cellular energy source which is used for active transport of additional solutes
IPR008004	Uncharacterised protein family UPF0503	4	Plant chloroplastic proteins
IPR017930	Myb domain	15	Nuclear DNA-binding protein
IPR000109	Proton-dependent oligopeptide transporter family	10	A group of energy-dependent transporters involved in the intake of small peptides
IPR006015	Universal stress protein A	5	Predicted to be related to the MADS-box proteins and bind to DNA, expresses in *E. coli* under growth arrest condition
IPR017949	Thaumatin, conserved site	5	Involved in systematically acquired resistance and stress responses in plants
IPR000315	B-box-type zinc finger	6	Proteasome-mediated degradation
IPR005516	Remorin, C-terminal	5	Plant-specific plasma membrane-associated proteins roles possible as component of membrane/cytoskeleton
IPR029962	Trichome birefringence-like family	7	Impact pathogen resistance, freezing tolerance, and cellulose biosynthesis
PF14416	PMR5 N terminal Domain	7	Sugar binding
IPR013525	ABC-2 type transporter,	7	Uses hydrolysis of ATP to energies diverse biological process, are involved in the export or import of a wide variety of substrates ranging from small ions to macromolecules
IPR000368	Sucrose synthase,	4	Synthesis of sucrose

### KEGG analysis revealed alteration in wound-responsive metabolism

The integration of genomic, biochemical and systemic function information can be found at KEGG (Kyoto Encyclopedia of Genes and Genomes), which is a biological system and resource management database (Kanehisa et al., 2012). We retrieved UNIPROT protein IDs for the DEGs identified in defoliated *B. distachyon* by using the PANTHER web server (Mi, Muruganujan & Thomas, 2013). Subsequently these protein IDs were screened for KEGG pathway annotation; the corresponding significance levels were calculated by using hypergeometric test/Fisher’s exact test, while FDR correction was achieved by using Benjamini & Hochberg (1995) method provided by the KOBAS web server (Mao et al., 2005). A total of 16 significant KEGG pathway categories were identified, with the criterion that at least four DEGs were represented (Fig. S4). Genes corresponding to biosynthesis of secondary metabolites (KEGG ID: 1110), glycolysis (KEGG ID: 10), and metabolic pathways (KEGG ID: 1100) were overrepresented in the transcriptome dataset (Table 2; Table S4). These results are consistent with wounding-induced transcriptional activation of several plant signaling cascades including oxidative phosphorylation, plant hormone signal transduction, protein processing in ER, and shikimic acid metabolism, presumably to activate the response to wounding stress and to prepare the plants for secondary metabolite synthesis and for mobilization of energy (Table S4).

### Overrepresentation of a group of transcription factors in DEGs

Gene regulation by TFs is common and important for cellular response to biotic and abiotic stress conditions. To better understand the molecular mechanism underlying *B. distachyon* response to defoliation, we searched for TFs among the DEGs and identified their gene families according to the data base Plant TFDB (Guo et al., 2008). Out of the 1,263 DEGs (≥2-fold change), 95 are TF belonging to 23 TF family, with MYB, bHLH, C2H2, WRKY, MYB-related, ERF, NAC, and trihelix families containing more than five DEGs in the transcriptome (Fig. S5). Further, the 1,263 DEGs (≥2-fold change) were subjected to transcription factor enrichment analysis in Plant TFDB, which finds TFs with significant over-represented targets in the input genes. The results revealed 36 families of 236 TFs, with MYB, NAC, ERF, bZIP, and WRKY as the top five most abundant TF families (Fig. S6), targeting 1,213 DEGs of mowed grass transcriptome. From this analysis it is inferred that TFs of MYB, bHLH, C2H2, WRKY, ERF, and NAC domain are major players of partial defoliation- induced plant response. The expression of TF genes such as MYB, WRKY, and AP2-domain family has been reported in response to wounding (Cheong et al., 2002), while partial defoliation of grasses leads to differential expression of TFs regulating hormonal homeostasis (such MYB, bHLH, ERF, WOX, and HD-ZIP), of ion-binding TFs (C2H2, DBB, C3H, FAR1, Co-like, and ZF-HD), as well as of tissue specific (Myb related and LBD) and other transcription regulating TFs (Table S5). Therefore, compared to wounding, partial defoliation displayed broader impacts on plant transcriptional regulation of TFs and probably their downstream targets.

**Figure 3:**
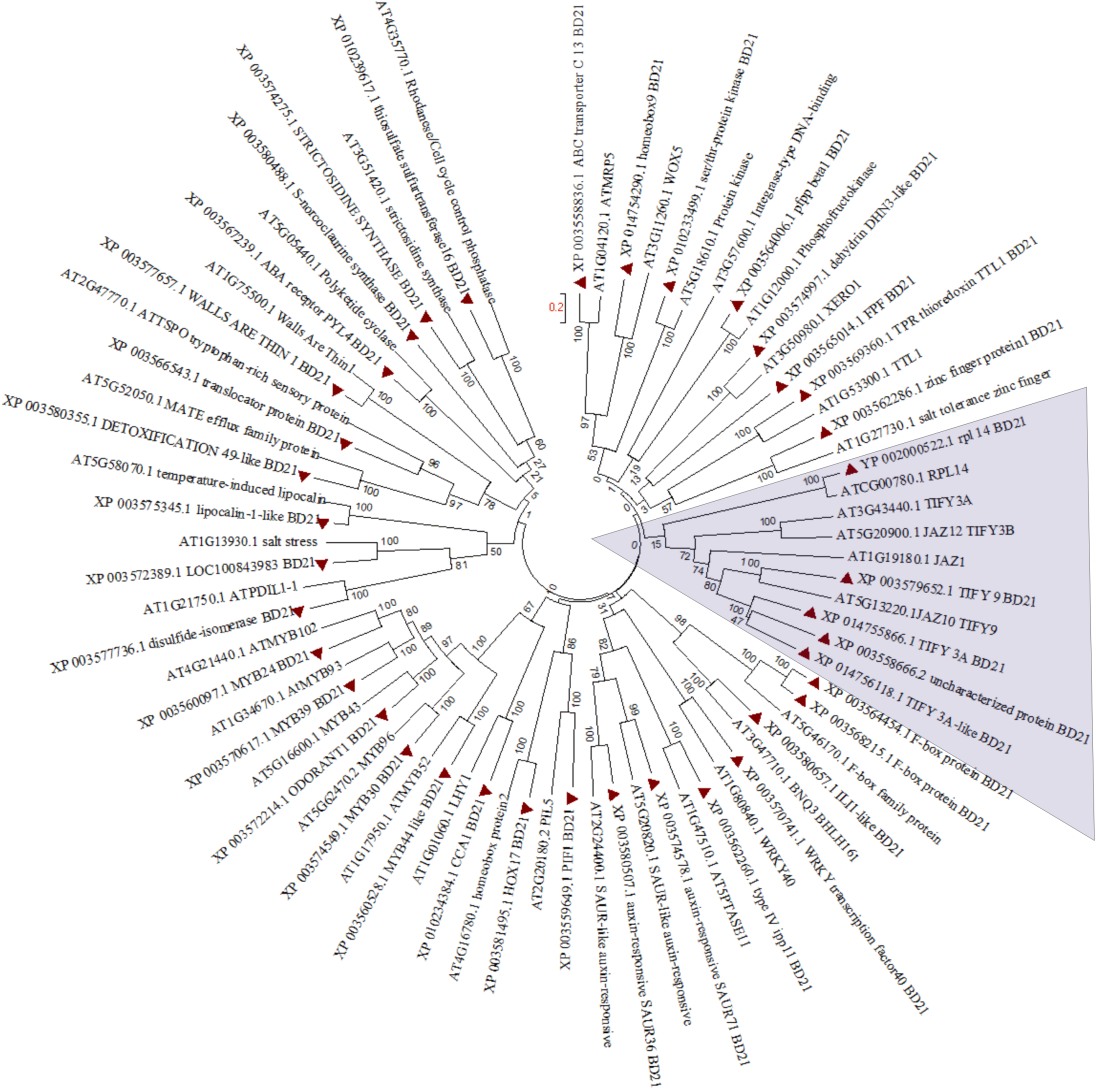
Phylogenetic tree for hormone responsive DEGs in *B. distachyon* treated with mowing and their homologs in *Arabidopsis thaliana*. Shaded area shows genes belongs to JA signaling. Tree was constructed from clustal W alignment file of proteins from *B. distachyon* and *A. thaliana* by neighbor joining method with 1,000 bootstrap using Mega 6.0.

In order to find out partial defoliation-triggered *B. distachyon* DEGs that are conserved among different species, the orthologs for these DEGs were searched in *Arabidopsis thaliana*. Subsequently, the hormone-responsive genes identified in GO analysis study were subjected to clustering (Fig. 3). Most of the genes showed one to one clustering with its counterpart in *A. thaliana*, such as TFs (like MYB, WRKY, bHLH, and homeobox domain), kinases, synthesase, and other enzymes. As can be seen in Fig. 3 and Fig. S7A, the JA related proteins (wound stress responsive protein) from two species clustered separately, revealing the divergence of grass proteins from their counterpart in Arabidopsis. This pattern may indicate a difference in stress adaptability between *B. distachyon* and Arabidopsis because JA is known to mediate plant stress response and because grasses are more often subjected to wounding than Arabidopsis. Similarly, some extent of divergence can be observed among genes from two species involved in ion homeostasis (Fig. S7B).

### Cutting the aerial portion mildly decreases *B. distachyon* root rhizosphere acidification

**Figure 4:**
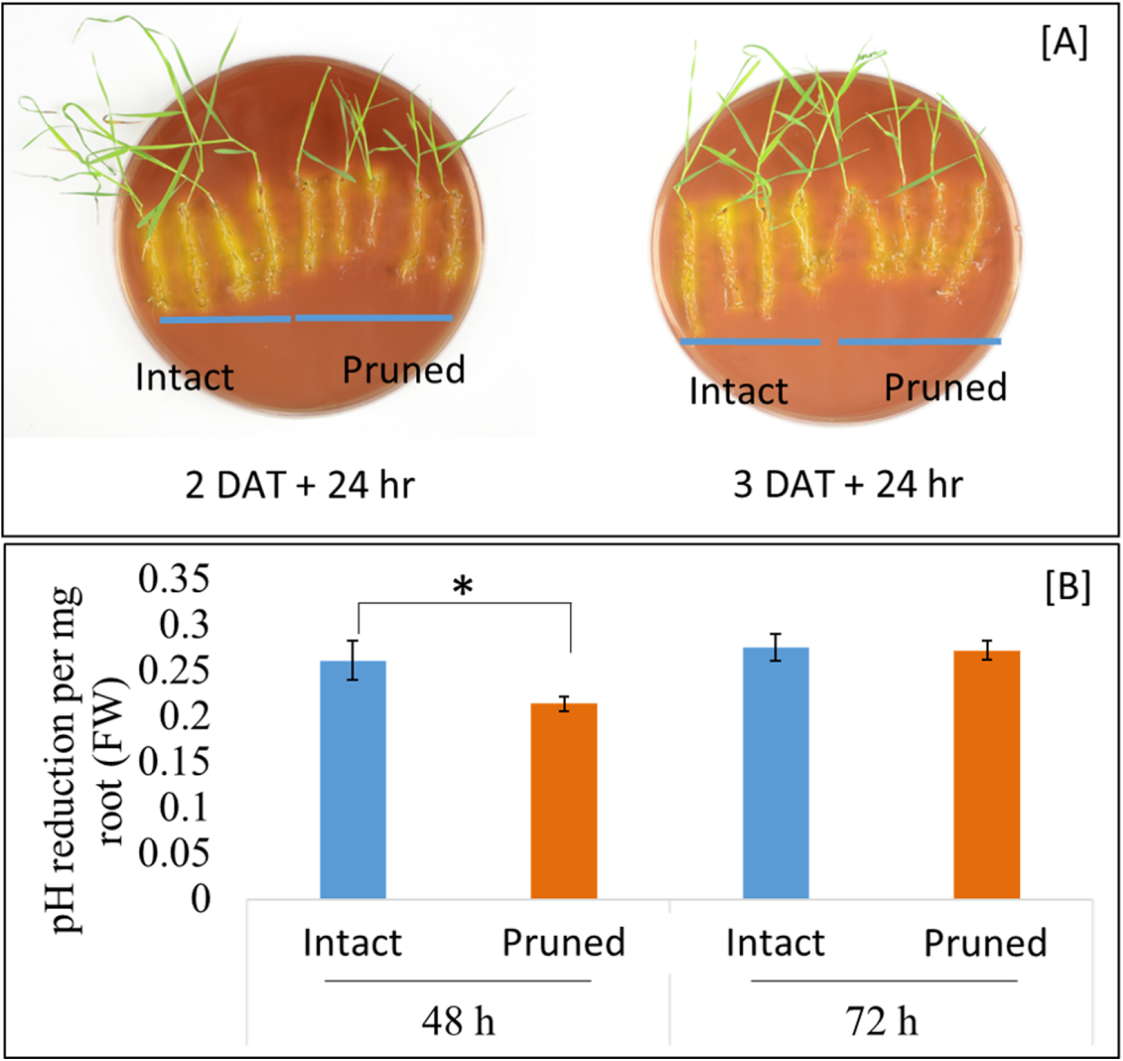
Rhizosphere acidification by *B. distachyon* roots with and without mowing treatment. (A) The pH indicator bromocresol purple shows the acidity of rhizosphere, with stronger yellowish indicating lower pH. Plants at 2 or 3 days after treatment were transferred to medium with pH indicator for 24 h before imaging, (B) quantification of change in pH at 2 and 3 days after treatment for control and treated plants. Values are mean ± SD, *n* ≥ 6 biological replicates. Asterisks indicate statistical significance, student *t*-test, *p* < 0.05.

Soil pH at the vicinity of roots, that is, rhizosphere, is an important determinant of the mobility of many nutrient elements. Roots releases proton (H^+^), in exchange of cations, through root plasma membrane H^+^-ATPase. Our RNAseq analysis identified three root ATPase-related DEGs (Table S3). To examine the effect of partial defoliation on root rhizosphere acidification, both pruned and intact plants were transferred to new growth medium with the pH indicator bromocresol purple, which exhibits a yellowish color when pH is below 5.2. As shown in Fig. 4, *B. distachyon* roots were surrounded by yellowish color independently of the partial defoliation treatment, although the pruned plants displayed a weaker intensity of the yellowish color in the rhizosphere compared to the intact plants. Such a mild reduction in rhizosphere acidity was similarly observed with *B. distachyon* plants at 1, 2, and 3 DAT (Fig. 4; Fig. S8), indicating the partial defoliation treatment quickly and continuously affected the capacity of roots in releasing H^+^ into the rhizosphere. It would be interesting to determine whether such a fine tuning of rhizosphere acidity contributes to the altered nutrient uptake in *B. distachyon* after partial defoliation.

### Partial defoliation regulates *B. distachyon* gene expression similarly in shoots as in root

**Figure 5:**
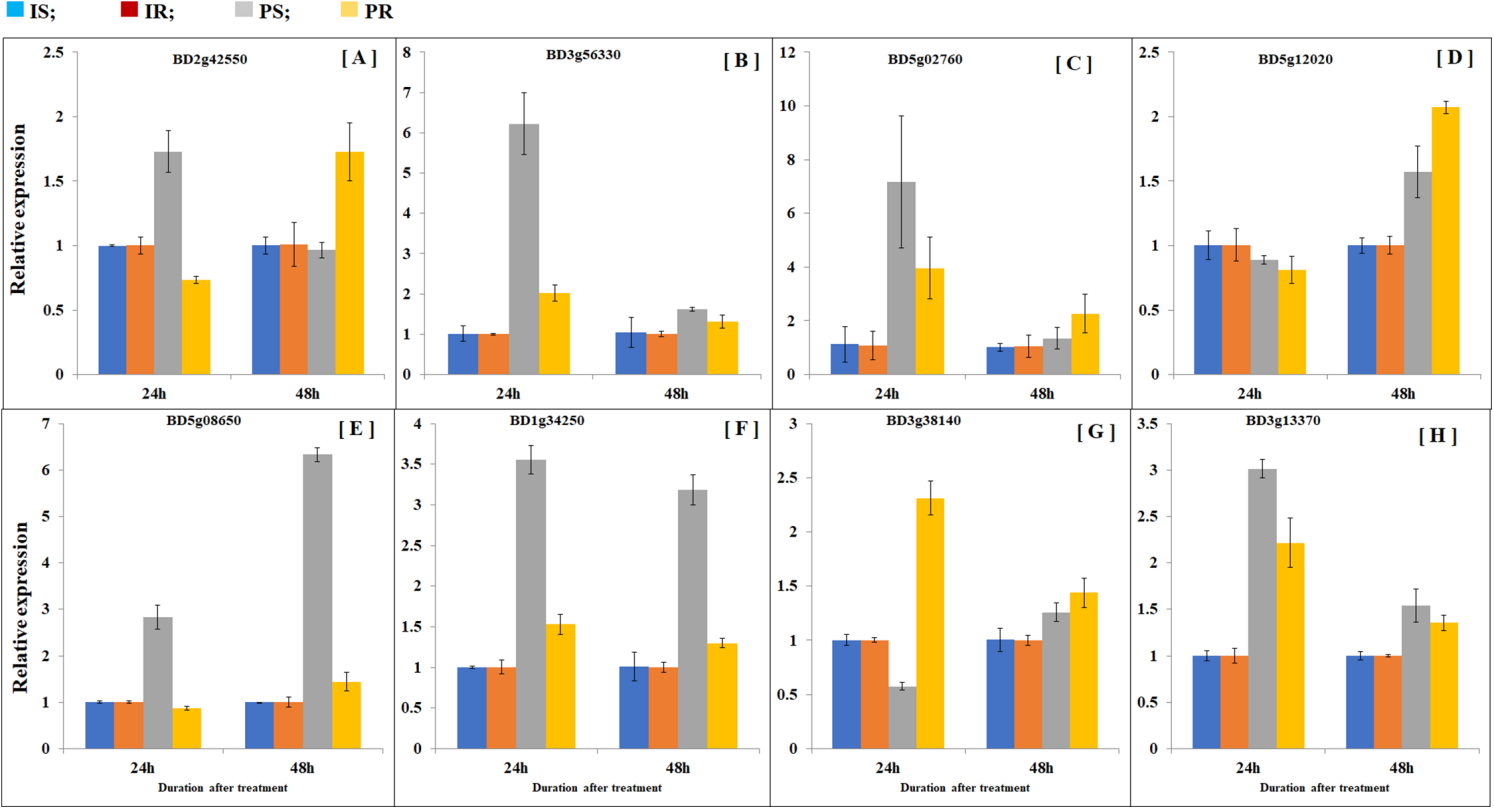
Comparison of gene expression patterns in shoot and roots. Gene expression levels of (A) BD2g42550, (B) BD3g56330, (C) BD5g02760, (D) BD5g12020, (E) BD5g08650, (F) BD1g34250, (G) BD3g38140 and (H) BD3g13370 were quantified by quantitative real-time PCR. Values are mean ± SD, *n* = 3 biological replicates. Samples were harvested at 24 and 48 h after the mowing treatment. CS, control shoots; CR, control roots; TS, treated shoots; TR, treated roots.

In order to compare transcription patterns between *B. distachyon* roots and shoots, we extended gene expression analysis by doing quantitative real-time PCR using both roots and shoots. Plant samples were collected at 24 and 48 h after partial defoliation treatment to disclose early transcriptional regulation. A group of eight genes were examined including Bradi5g02760 (probable inorganic phosphate transporter 1–4), Bradi3g56330 (sodium-coupled neutral amino acid transporter 1), Bradi2g42550 (metal ion binding protein), Bradi5g12020 (sugar transport protein 5-like), BD3g13370 (pyruvate decarboxylase 2), BD5g08650 (aba receptor 8), BD1g34250 (two-component response regulator orr3 isoform x4), and BD3g38140 (acc oxidase). As expected, all the examined genes displayed similar patterns as observed in RNAseq (Fig. 5; Table S6). Besides, partial defoliation-triggered gene expression patterns in roots were similarly observed in shoots in general (Fig. 5; Table S6), indicating that root RNAseq results are representative of the whole *B. distachyon* plants, in addition to providing organ-specific accuracy of transcriptional regulation patterns. Consistent with elevated phosphorus levels in roots and shoots (Fig. 1), partial defoliation increased gene expression of Bradi5g02760, which is a putative transporter of inorganic phosphate, at both 24 and 48 h after the treatment and in both roots and shoots (Fig. 5). Therefore these observations further support the conclusion that, during recovery from partial defoliation, *B. distachyon* plants elevate root uptake of nutrient elements such as Fe and P, at least partially, through transcriptional regulation.

## Discussion

**Figure 6:**
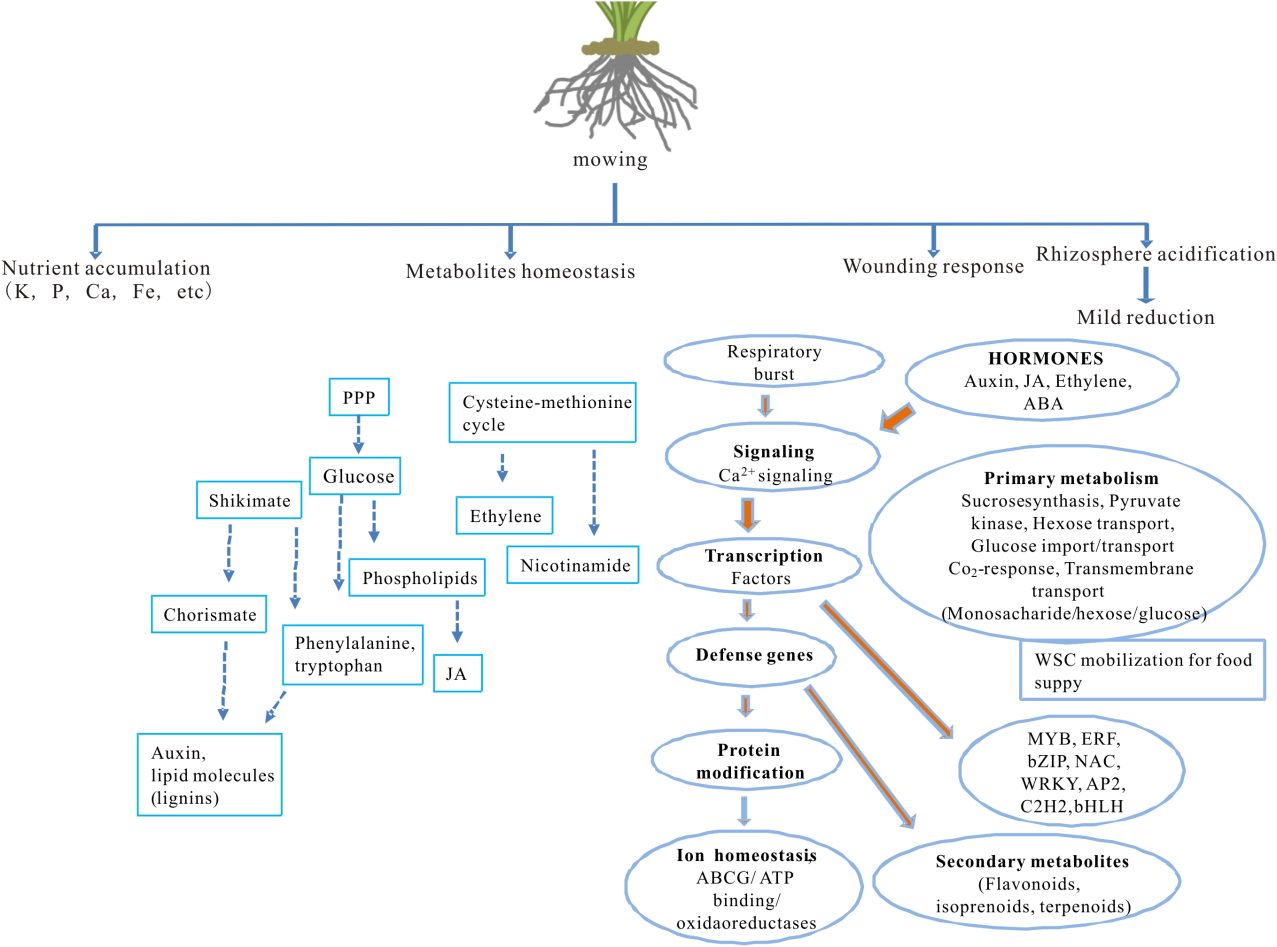
A proposed model showing the effects of partial defoliation on *B. distachyon*. The model has included alterations in nutrient ion accumulation patterns, transcriptional regulation on secondary metabolite homeostasis and on wounding responses, as well as root rhizosphere acidification.

The impact of partial defoliation on grass, especially regarding to root ion homeostasis and transcriptome, has been unclear. This study reveals how partial defoliation alters ion accumulation levels in a shoot- and root-specific manner, as well as partial defoliation-induced transcriptional reprogramming on a whole-genome scale (Fig. 6; Fig. S4), thereby providing a solid base for further in-depth elucidation of the underlying molecular mechanisms in grass species and possibly also other monocot plant species.

Our results showed that Fe levels were very high in roots, which implies that Fe has other roles that for photosynthesis or just stores in root for photosynthesis. Longnecker & Welch (1990) and Curie & Mari (2017) reported respectively that root apoplastic storage of iron were a conserved mechanism for plant responses to iron deficiency. Iron is also important for electron transfer reactions and is critical for biosynthesis of photosynthesis apparatus (Rochaix, 2011). Together these ion accumulation patterns demonstrate a strong impact by partial defoliation on *B. distachyon* ion homeostasis, implying potential roles of certain metal ions, such as iron which is known to be critical for producing photosynthetic apparatus, in the recovery process after the partial defoliation treatment.

Our results indicate that roots are the major source of iron storage in Bd21. The quantification of nutrients in roots and shoots of *B. distachyon* provide an overview of total ion flux in response to partial defoliation. Analyses of shoot and root samples at individual time points of interest further gives a more precise estimate of the plant ion homeostasis status, which possibly will be meaningful in predicting potential problems. Pruned resulted in higher levels of Na in shoots at both 4 and 7 DAT (Fig. 1E), implying an involvement of Na in the recovering of *B. distachyon* shoots from defoliation.

The ICP-MS analysis for plant samples at 4 and 7 DAT clearly showed that the pruned plants efficiently mobilized different essential nutrients such as phosphorus, potassium, iron, and magnesium, in roots and shoots in the process of recovering from the cutting treatment. Our results (Fig. 1D) are consistent with the fact that P is important for many principle biological processes such as DNA and protein synthesis; these results also suggest an important role of P in *B. distachyon* for recovering from defoliation.

In intact plants, K accumulated to similar levels in shoots and roots; meanwhile cutting the aerial portion caused a 29% increase in root K levels at 4 DAT, although K levels in pruned roots at 7 DAT fell back to a level that was similar to that in intact roots (Fig. 1C), indicating a temporary need for extra K in *B. distachyon* to cope with defoliation. The higher levels of Ca in both shoots and roots indicate an important role of Calcium in *B. distachyon* response to defoliation.

Our transcriptional investigation detected an elevation of some JA-related genes (Table S3) that are wounding-responsive, such as DEGs related to ascorbate peroxidase and transporters (Suza et al., 2010), phospholipase (Wang et al., 2000), peroxidases (Minibayeva, Beckett & Kranner, 2015), and cytokinin related genes (Schafer et al., 2014), cytochrome p450 (Koo, Cooke & Howe, 2011; Pinot & Beisson, 2011), monooxygenases (Kong et al., 2016). In Fig. S7, jasmonic responsive genes along with their corresponding homologs from Arabidopsis are present in a cluster, which consists of TFs, secondary metabolite proteins, phosphatases and vascular protein sorting genes. A recent study suggested hormone crosstalk at metabolic level during wound stress (Zhang et al., 2016). Consistently, different hormone responsive genes were identified in this transcriptome study and the corresponding homologs in Arabidopsis were clustered (Fig. 2). The presence of genes related to auxin, JA, gibbrelin, and ET further highlights the crosstalk of hormones in *B. distachyon* in response to the partial defoliation treatment (Tables S2 and S3). These transcriptional regulation patterns likely reflect the strong impact of partial defoliation, that is, removal of photosynthetic organ, on plant energy production and relocation. In addition, GO terms like plant-type vacuole, membrane, intrinsic and integral component of membrane were noticeable in the cellular component category. As partial defoliation activates the secondary metabolite production in response to wounding stress which may be toxic to plants. In such a case, excessive levels of these compounds may trigger in planta transportation to the apoplast or to specific organelles such as vacuoles (Nobukazu, 2016). In addition, the GO terms “sulfate assimilation” and “anion homeostasis” were also enriched, indicating that plants also adjust anion homeostasis to cope with the altered metal ion accumulation in response to the partial defoliation treatment. Cysteine and methionine metabolism pathway genes were found to be up-regulated, indicating possible activation of the related biological processes such as sulfate assimilation, ET production, and metabolism of secondary metabolites like nicotinamine (Halimaa et al., 2014). At the same time, genes corresponding to gluconeogenesis/glycolysis were down-regulated, indicating conservation of energy in the form of glucose, which may be utilized for JA synthesis that is required for initiation of the defense signaling cascade (Halimaa et al., 2014).

Differentially Expressed genes related to carbohydrate and energy generation are highly enriched, including at least 28 GO terms (Table S1) and eight KEGG pathways. These patterns indicate that the pruned plants tend to conserve energy and mobilize it for new organ development, which is an important process after defoliation of forage crops (Prud’homme et al., 1992; Donaghy & Fulkerson, 1997; De Visser, Vianden & Schnyder, 1997; Morvan-Bertrand et al., 2001). Wounding in plants also leads to secondary metabolites production, which is mediated by signaling-molecules such as ROS, ET, and JA (Jacobo-Velázquez, González-Agüero & Cisneros-Zevallos, 2015). The identification of DEGs related to isoprenoid, terpenoid, alcohol-responsive genes, and lipid pathway genes (Table S1), indicates the generation of secondary metabolites in *B. distachyon* plants in response to partial defoliation. In addition, DEGs in the shikimate pathway and in the nicotinamide nucleotide metabolic process were also identified; the former is involved in producing intermediates of secondary metabolites (Taiz & Zeiger, 2006), while the latter intacts crucial functions of carbon primary and secondary metabolism (Pétriacq et al., 2013). Thus, these patterns further indicate an induced accumulation of secondary metabolites in *B. distachyon* after mechanistic damage. The DEGs obtained were further subjected to their protein class analysis for the corresponding genomes based on InterPro and Pfam protein database classification in STRING v10 software (Szklarczyk et al., 2015).

Among the identified DEGs in defoliated *B. distachyon* are a group of TF, such as MYB, WRKY, NAC, bHLH, and ERF (Fig. 6). Wounding induces expression of TF genes such as MYB, WRKY, and AP2-domain family genes (Cheong et al., 2002). The study by Rajendran et al. (2014) demonstrated critical roles of NAC and WRKY transcription factors in plant responses to mechanical wounding and herbivory, while certain bHLH and NAC transcription factors control the balance between plant vitality and survival during wounding (Chen et al., 2016). In addition to NAC, WRKY, and bHLH, partial defoliation also altered gene expression of some ion binding TFs like C2H2, DBB, C3H, FAR1, Co-like, and ZF-HD.Moreover, tissue specific TFs like Myb-related and LBD are expressed in response to partial defoliation apart from wound induced TFs (Table S5). The crosstalk between ET and jasmonate pathways has been well-documented, and it has been proposed that ERFs play critical roles in integrating signals from the two pathways during plant defense (Lorenzo et al., 2003; Walley et al., 2007). Overrepresentation of these differentially regulated TF thus suggest a prominent role of transcriptional regulation in *B. distachyon* pruned by cutting the aerial portion.

## Conclusion

This study reveals how partial defoliation alters ion accumulation levels in shoots and roots, as well as partial defoliation-induced transcriptional reprogramming on a whole-genome scale, thereby providing insight into the molecular mechanisms underlying the recovery process of grass after partial defoliation. Compared to the intact plants, roots of *B. distachyon* with defoliation showed 38% and 19% increases in Fe levels at 4 days and 7 DAT, respectively, whereas Fe levels in shoots were similar between the pruned and the intact plants. Similar to Fe, accumulation of Calcium (Ca) was increased in roots by cutting the aerial portion. At 4 and 7 DAT, the levels of Ca in pruned roots were 13% and 22% greater than those in intact roots. Partial defoliation mildly decreases *B. distachyon* root rhizosphere acidification and induces transcriptional alterations on plant ion homeostasis, hormones, primary and secondary metabolites, and wound stress.

## Supplemental Information

10.7717/peerj.7102/supp-1 Supplemental Information 1 GO enrichment analysis of the overlapping DEGs. GO term classification including biological process, cellular component and molecular function.

10.7717/peerj.7102/supp-2 Supplemental Information 2 Information for DEGs was classified into different gene functional groups. Gene grouping including major grouping and functional grouping.

10.7717/peerj.7102/supp-3 Supplemental Information 3 Information for DEGs related to response to hormone was further classified into different gene functional groups. DEGs related to hormone respnse classified into different groups like ABC transporter, ATPase, Cytochrome etc.

10.7717/peerj.7102/supp-4 Supplemental Information 4 KEGG pathway enrichment analysis of the overlapping DEGs was performed. Details of KEGG pathway enrichment analyisis.

10.7717/peerj.7102/supp-5 Supplemental Information 5 Information for DEGs belonged to transcriptional factors was classified into different gene functional groups. Categorization of transcription factors.

10.7717/peerj.7102/supp-6 Supplemental Information 6 Comparison of gene expression patterns in shoot and roots between RNAseq and qRT-PCR data. qRT-PCR data of comparison of gene expression patterns in shoots and roots.

10.7717/peerj.7102/supp-7 Supplemental Information 7 Gene ontological classification of differentially expressed genes (DEGs) in *B. distachyon* treated by cutting the aerial portion, compared to the control. Twenty top ranked gene ontology (GO) based on p-value for biological, and molecular and all cellular processes are shown. See also supplementary Table 1 for all GO classification for different categories and grouping of GO classification according to their function in *B. distacyon*. Categories shown in graph are significant at p < 0.01.

10.7717/peerj.7102/supp-8 Supplemental Information 8 Gene Ontology information of the different expression genes.

10.7717/peerj.7102/supp-9 Supplemental Information 9 Depiction of differentially expressed genes with the ≥ 2-fold DEGs of *B. distachyon* for three biological replicates under different functional groups. Heat map showing differential expression of genes belonging to A; hormone response and regulation, B; response to abiotic stress and secondary metabolite, C; primary metabolism, D; membrane bound and ion homeostasis related. The normalized CPM value used in triplicate for control and treated sample to draw the heatmap using MEV 4.9.0.

10.7717/peerj.7102/supp-10 Supplemental Information 10 Depiction of different protein families present in the ≥ 2-fold DEGs of *B. distachyon* treated by cutting the aerial portion.

10.7717/peerj.7102/supp-11 Supplemental Information 11 KEGG (Kyoto Encyclopedia of Genes and Genomes) classification of the ≥ 2-fold DEGs identified in *B. distachyon* with mowing treatment compared to the control plants.

10.7717/peerj.7102/supp-12 Supplemental Information 12 Distribution of transcription factors (TF) gene families identified in the ≥ 2-fold DEG list.

10.7717/peerj.7102/supp-13 Supplemental Information 13 Distribution of transcription factors (TFs) identified in the ≥ 2-fold DEGs list of *B. distachyon* RNA seq data.

10.7717/peerj.7102/supp-14 Supplemental Information 14 Phylogenetic tree for genes of *B. distachyon* and their homologs in *Arabidopsis thaliana*. [A] Cluster of Jasmonic acid response genes; [B] Cluster of ion homeostasis genes. Tree was constructed from clustal W alignment file of proteins from *B. distachyon* and *A. thaliana* by neighbor joining method with 1000 bootstrap using Mega 6.0.

10.7717/peerj.7102/supp-15 Supplemental Information 15 Rhizosphere acidification by *B. distachyon* roots with and without mowing treatment. The pH indicator bromocresol purple shows the acidity of rhizosphere, with stronger yellowish indicating lower pH. Plants at 1 day after treatment were transferred to medium with pH indicator for 24 hours before imaging.

10.7717/peerj.7102/supp-16 Supplemental Information 16 The PCA graph showing replicate clustering patterns for RNAseq results.

10.7717/peerj.7102/supp-17 Supplemental Information 17 Rates of unique mapping in all RNAseq samples.

10.7717/peerj.7102/supp-18 Supplemental Information 18 Correlation coefficients between RNAseq biological replicates.

10.7717/peerj.7102/supp-19 Supplemental Information 19 The photo showed cutting range of partial defoliation on *B. distachyon* seedling.

10.7717/peerj.7102/supp-20 Supplemental Information 20 ICP data of ions before and after partial defoliation.

10.7717/peerj.7102/supp-21 Supplemental Information 21 PH values before and after partial defoliation.

## Additional Information and Declarations

### Data Availability

The following information was supplied regarding data availability: The sequence data have been deposited in NCBI’s Gene Expression Omnibus (GEO) and are accessible through GEO accession number GSE101500.
