# Supplementary figures and images for: Partial defoliation of Brachypodium distachyon plants grown in petri dishes under low light increases P and other nutrient levels concomitantly with transcriptional changes in the roots
Source: PeerJ. 2019 Jun 13;7:e7102. doi: 10.7717/peerj.7102 (PMC6571136; doi:10.7717/peerj.7102)

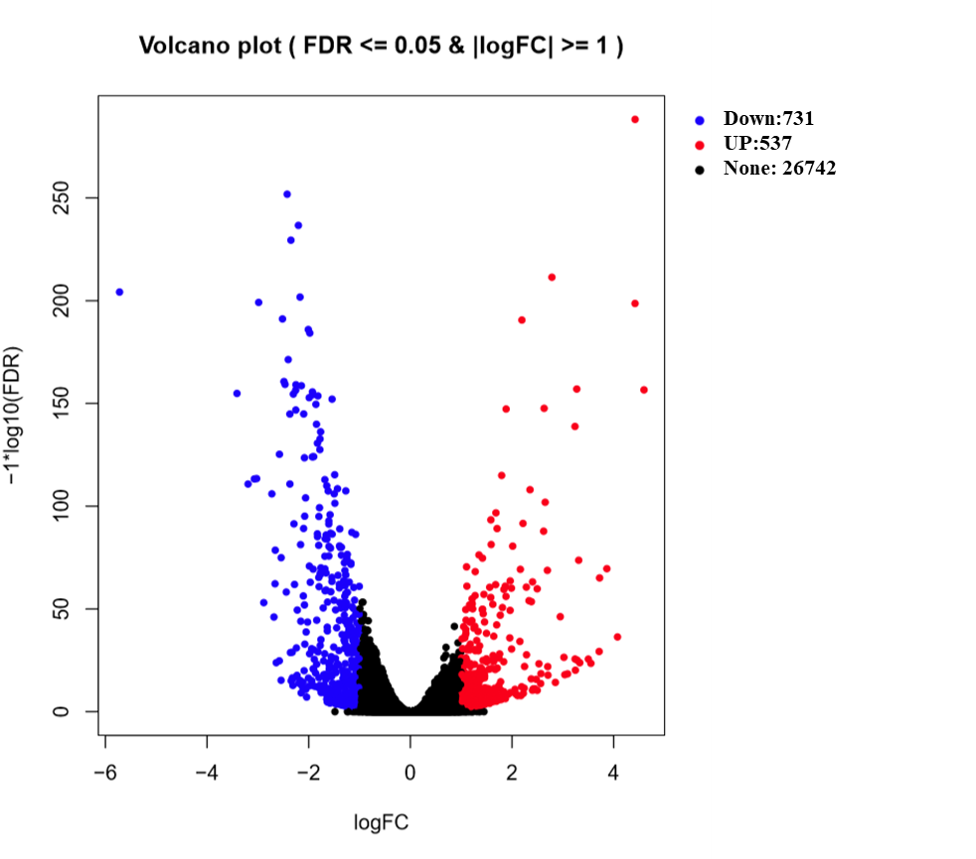

Supplement: Supplemental Information 7 — Twenty top ranked gene ontology (GO) based on p-value for biological, and molecular and all cellular processes are shown. See also supplementary Table 1 for all GO classification for different categories and grouping of GO classification according to their function in B. distacyon. Categories shown in graph are significant at p < 0.01. [file peerj-07-7102-s007.png]

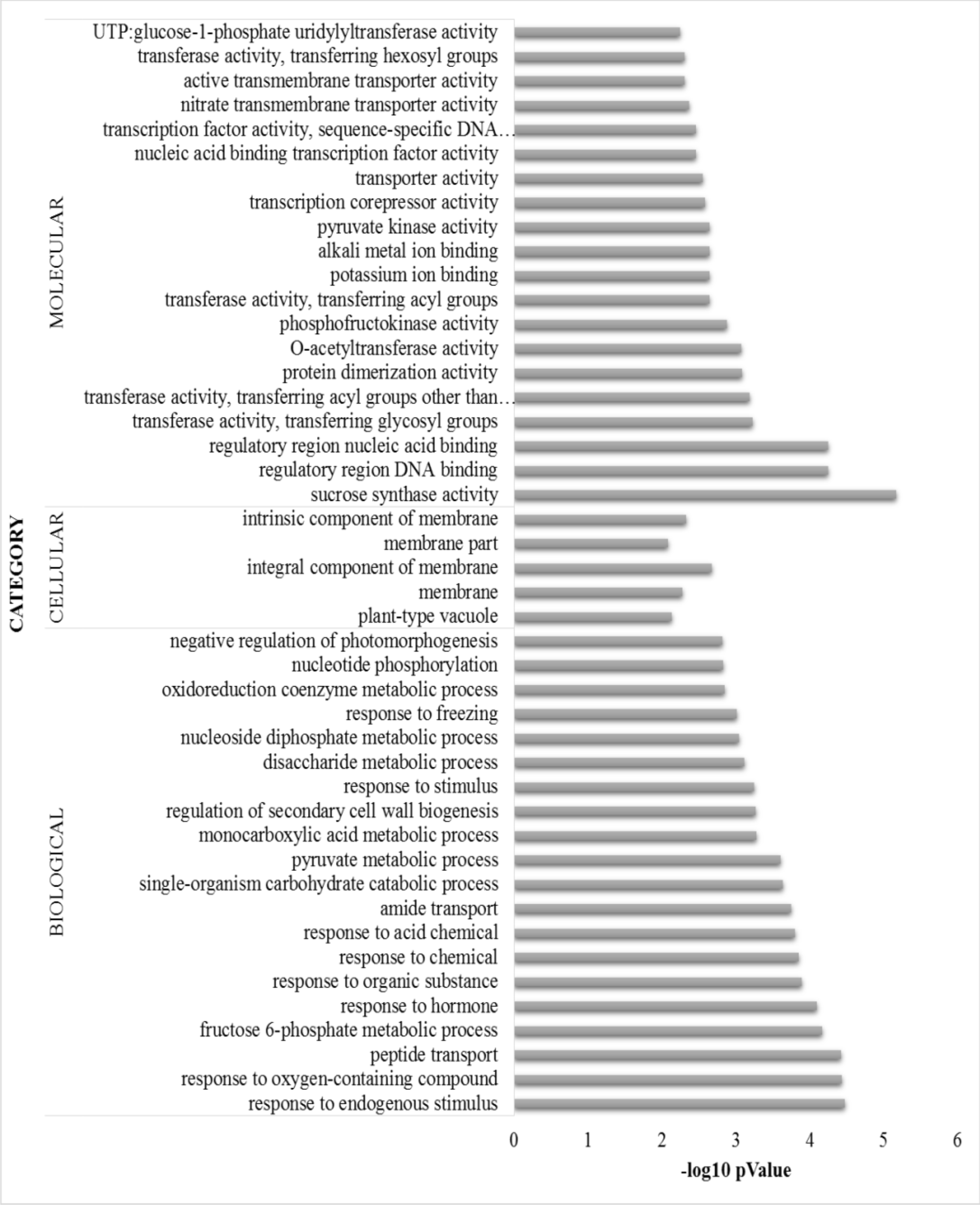

Supplement: Supplemental Information 8 [file peerj-07-7102-s008.png]

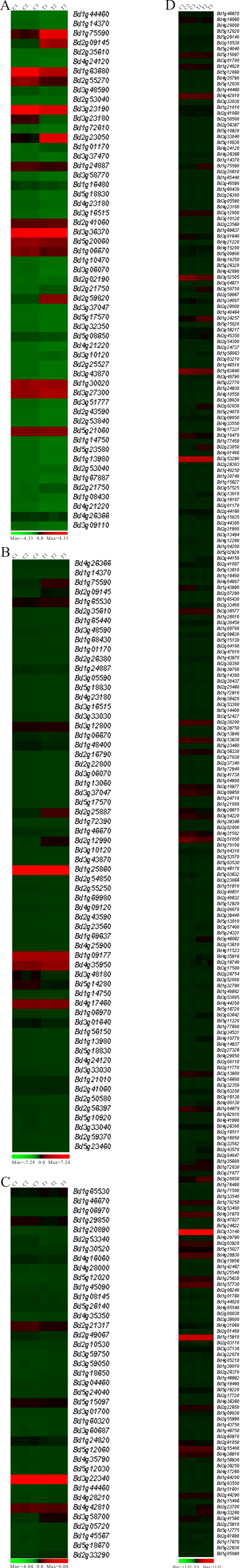

Supplement: Supplemental Information 9 — Heat map showing differential expression of genes belonging to A; hormone response and regulation, B; response to abiotic stress and secondary metabolite, C; primary metabolism, D; membrane bound and ion homeostasis related. The normalized CPM value used in triplicate for control and treated sample to draw the heatmap using MEV 4.9.0. [file peerj-07-7102-s009.png]

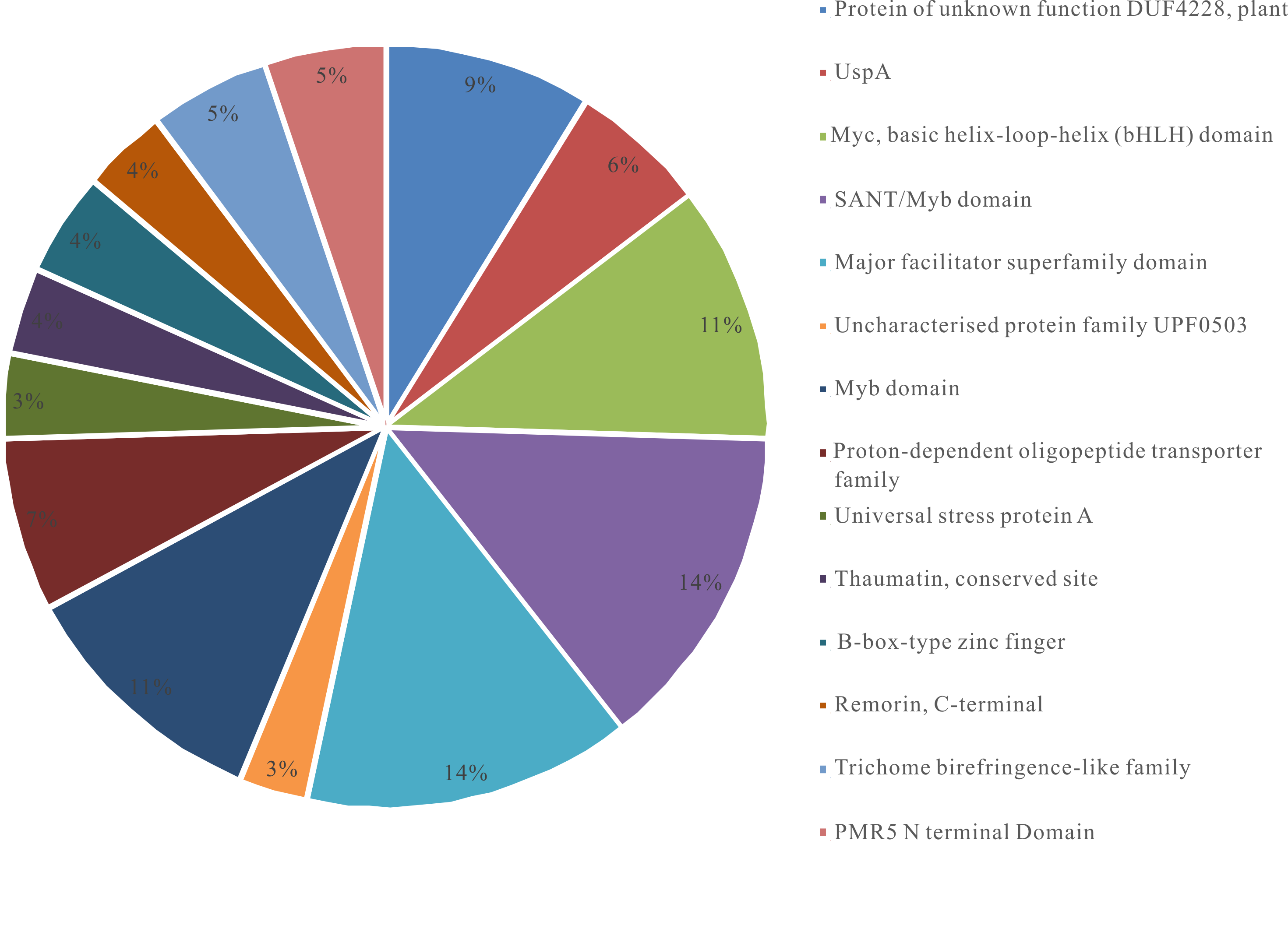

Supplement: Supplemental Information 10 [file peerj-07-7102-s010.png]

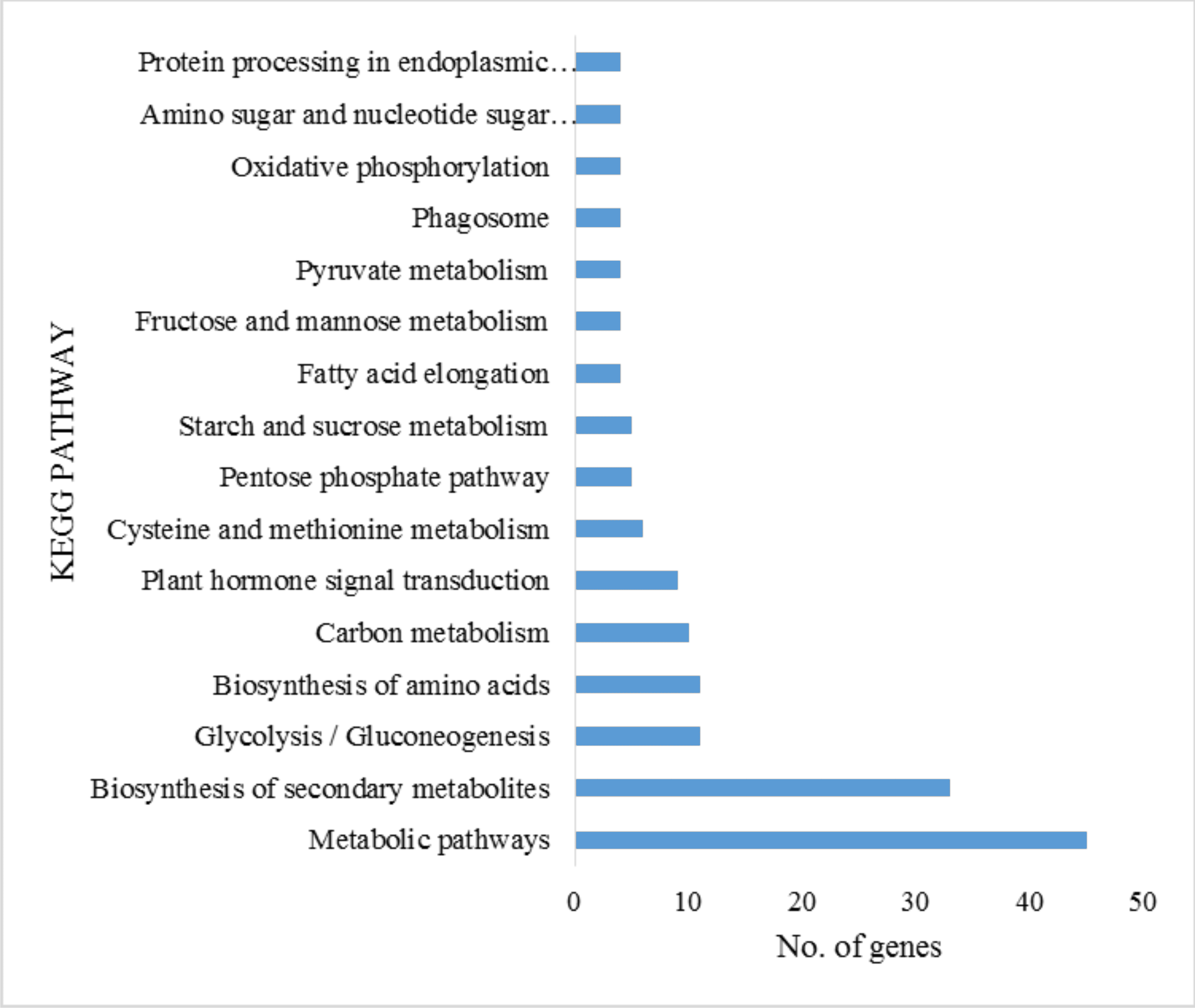

Supplement: Supplemental Information 11 [file peerj-07-7102-s011.png]

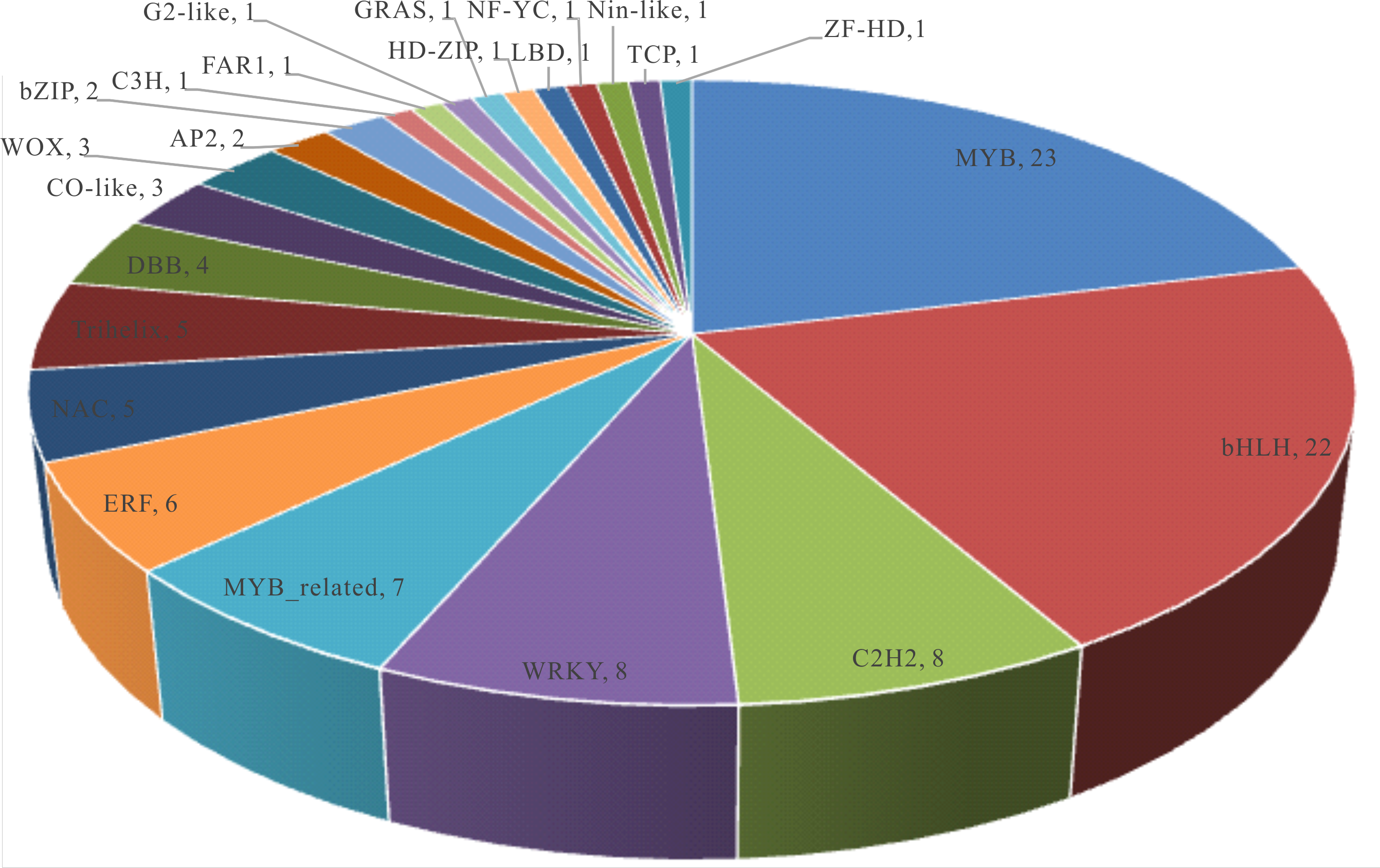

Supplement: Supplemental Information 12 [file peerj-07-7102-s012.png]

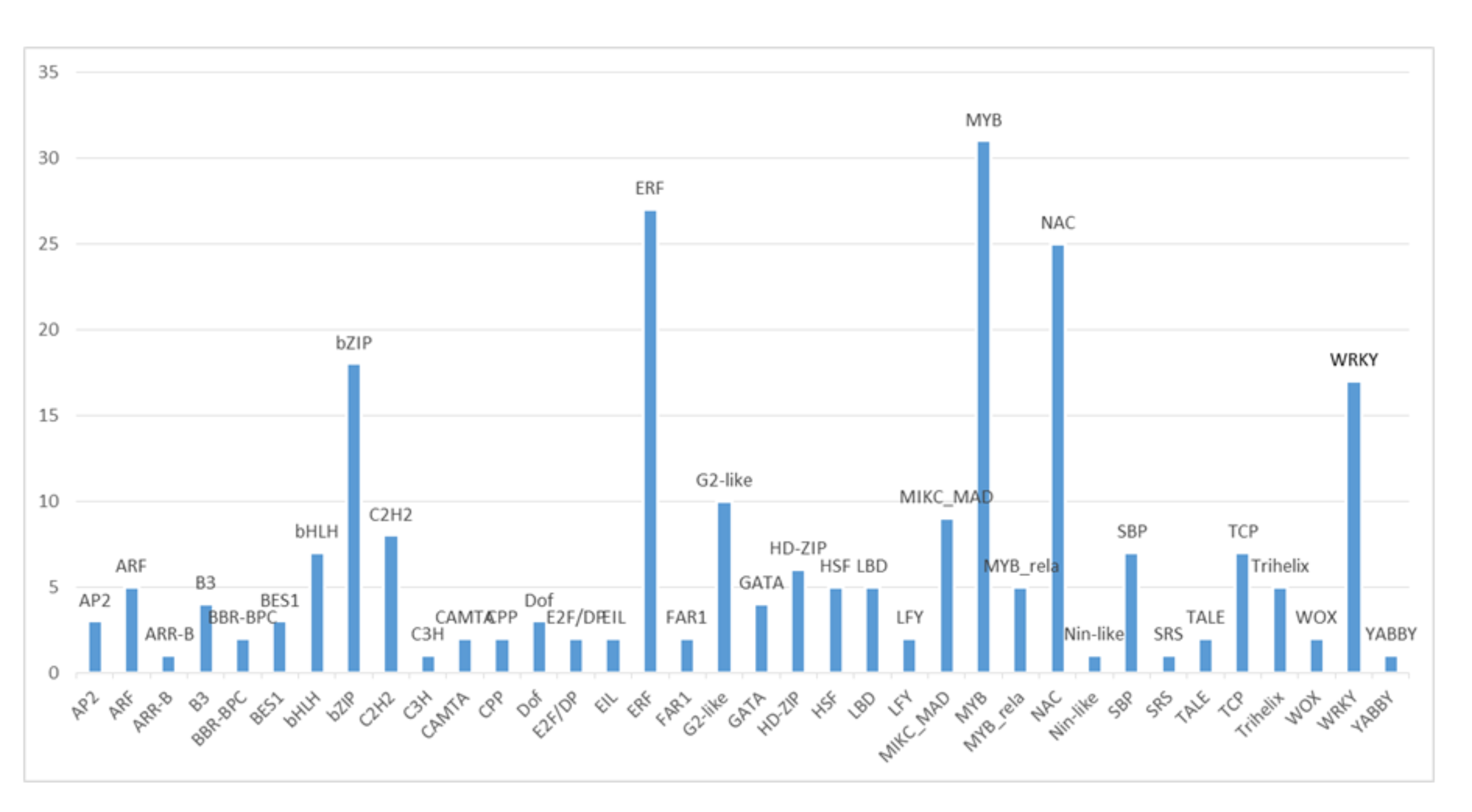

Supplement: Supplemental Information 13 [file peerj-07-7102-s013.png]

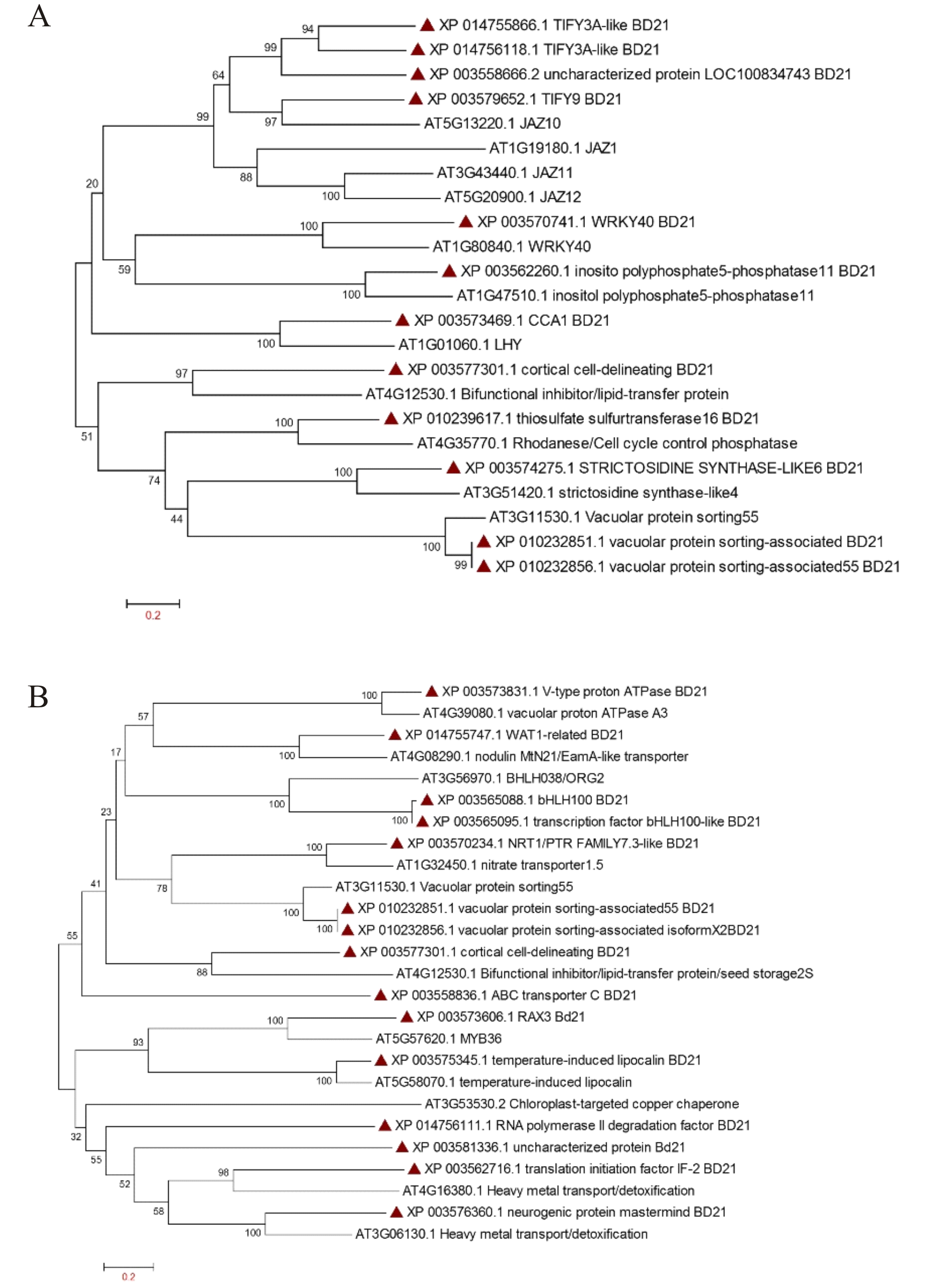

Supplement: Supplemental Information 14 — [A] Cluster of Jasmonic acid response genes; [B] Cluster of ion homeostasis genes. Tree was constructed from clustal W alignment file of proteins from B. distachyon and A. thaliana by neighbor joining method with 1000 bootstrap using Mega 6.0. [file peerj-07-7102-s014.png]

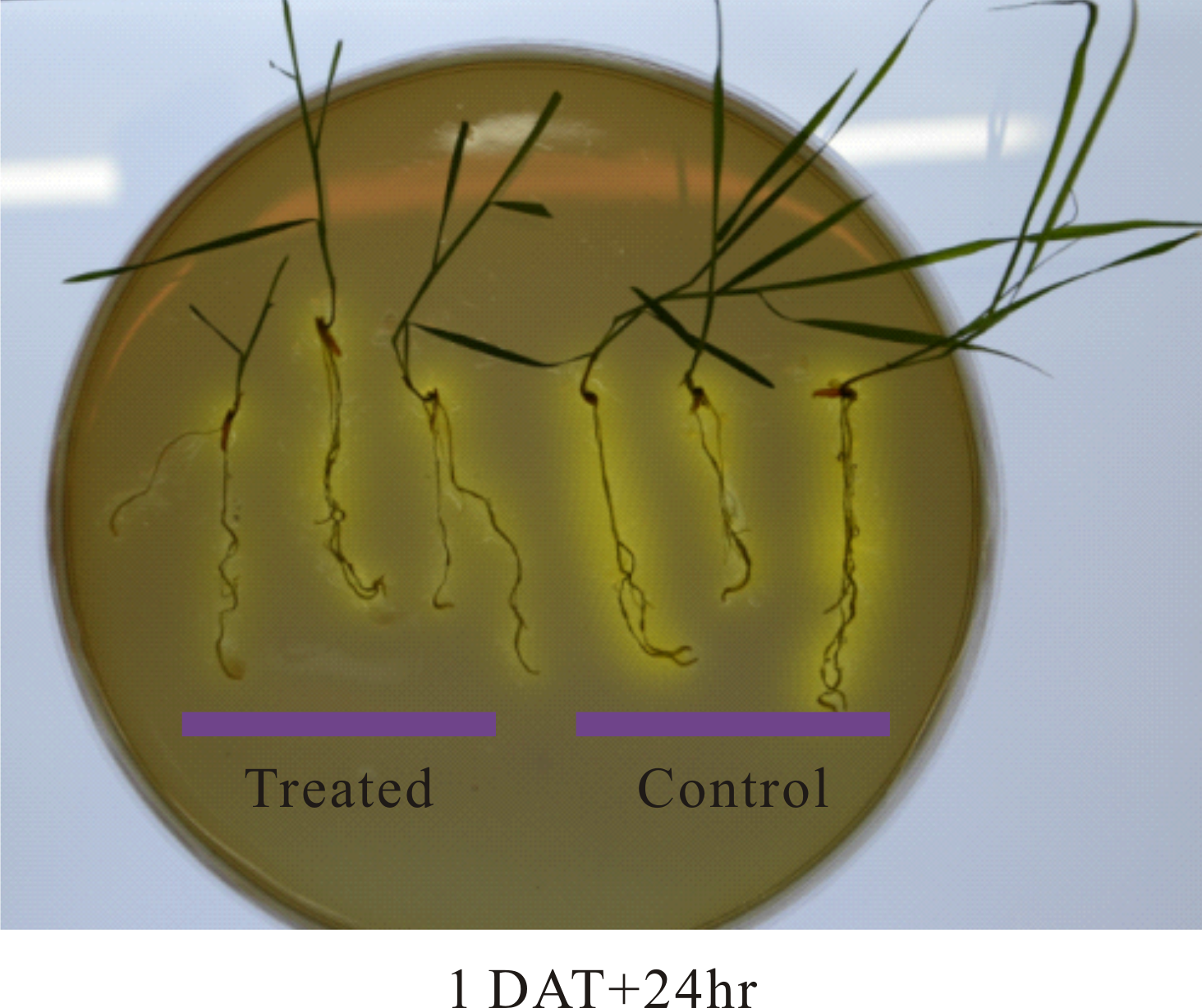

Supplement: Supplemental Information 15 — The pH indicator bromocresol purple shows the acidity of rhizosphere, with stronger yellowish indicating lower pH. Plants at 1 day after treatment were transferred to medium with pH indicator for 24 hours before imaging. [file peerj-07-7102-s015.png]

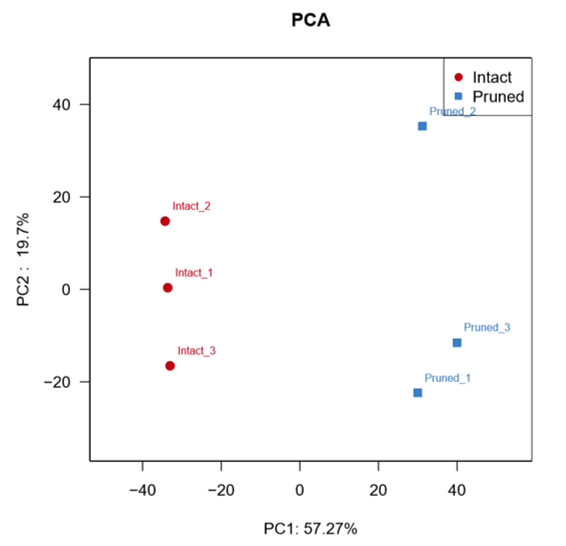

Supplement: Supplemental Information 16 [file peerj-07-7102-s016.png]

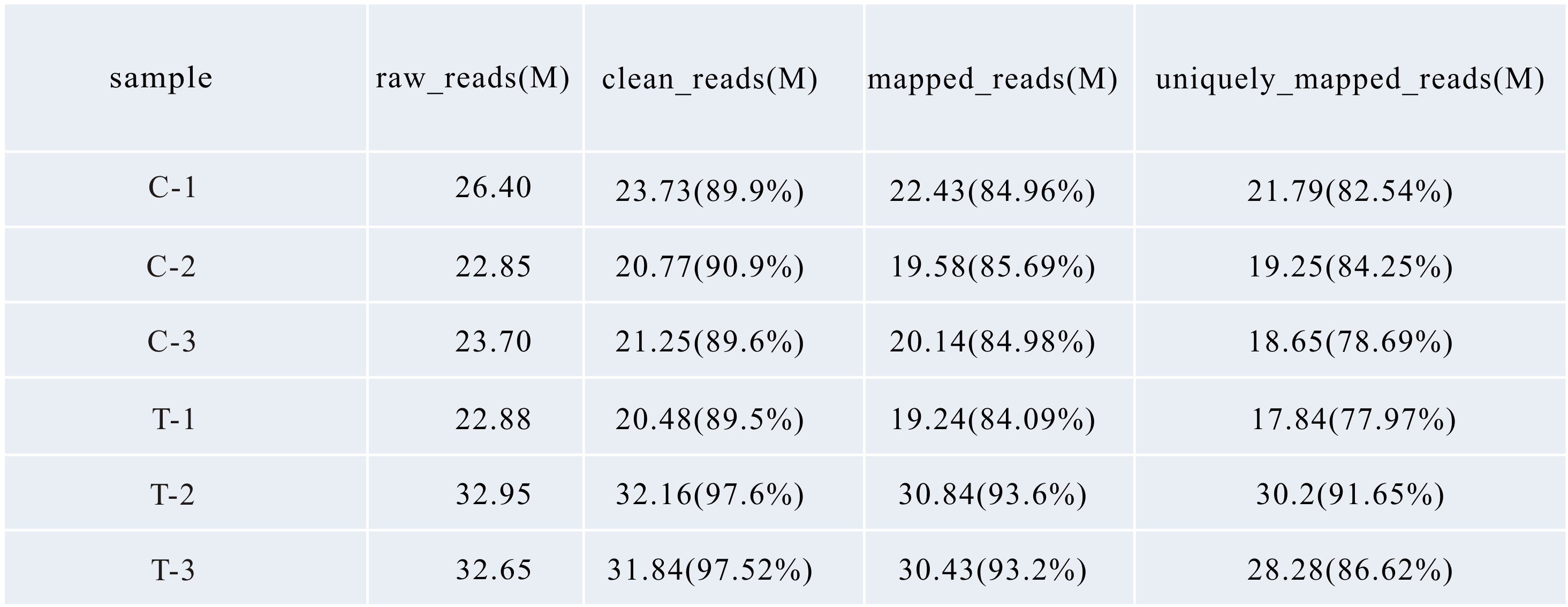

Supplement: Supplemental Information 17 [file peerj-07-7102-s017.png]

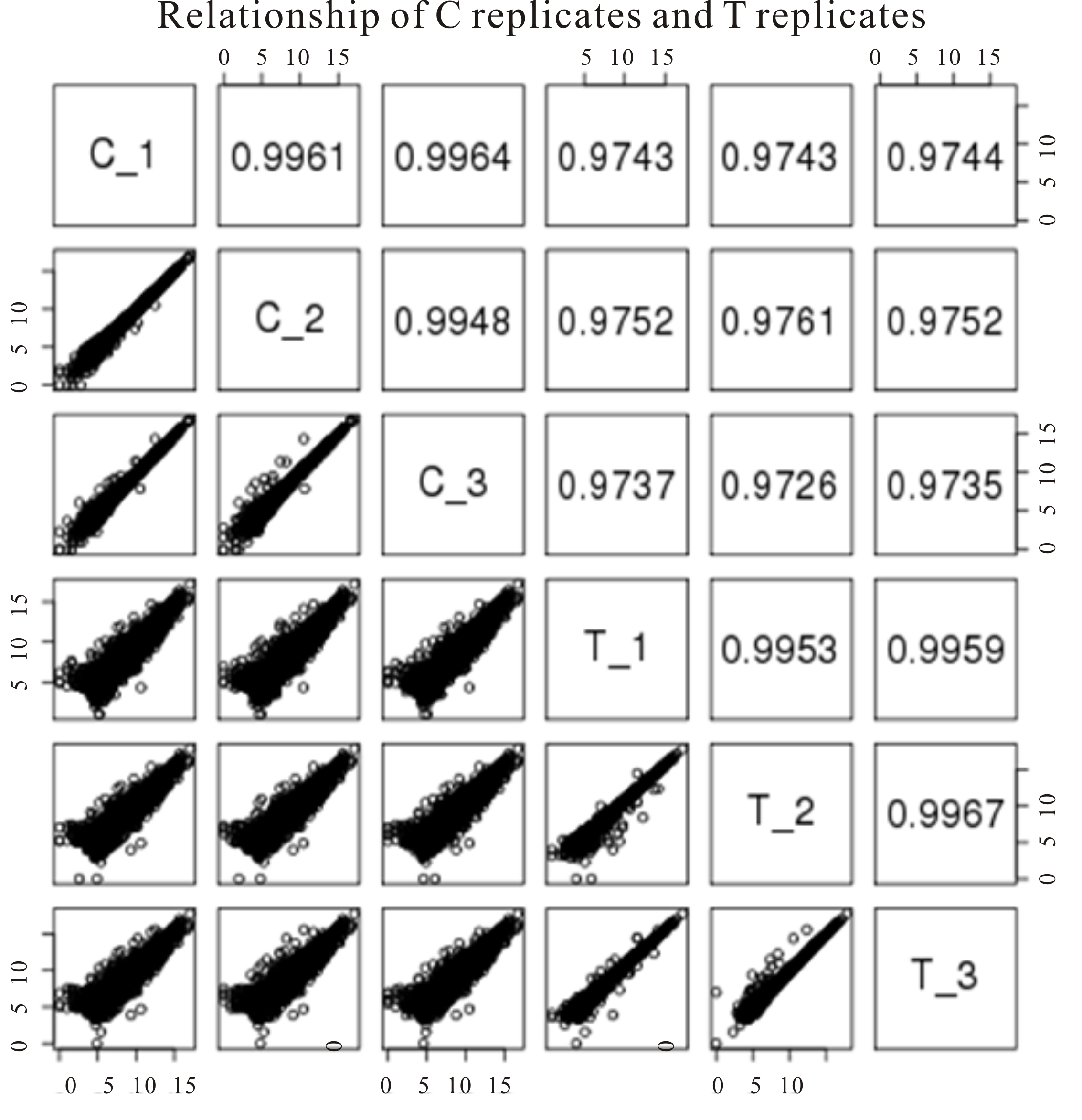

Supplement: Supplemental Information 18 [file peerj-07-7102-s018.png]

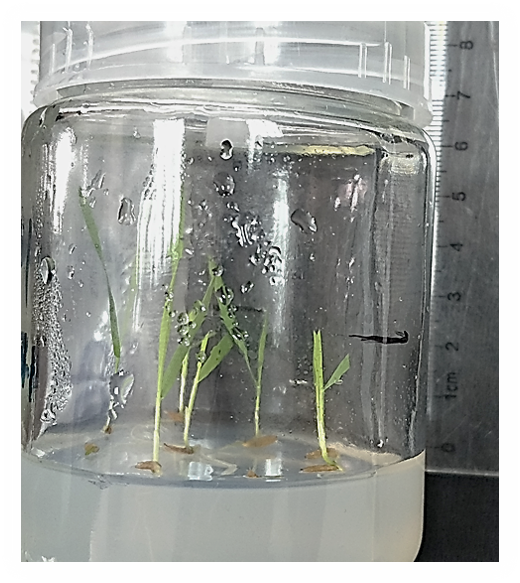

Supplement: Supplemental Information 19 [file peerj-07-7102-s019.png]
